# Dewlap colour variation in *Anolis sagrei* is maintained among habitats within islands of the West Indies

**DOI:** 10.1111/jeb.14002

**Published:** 2022-05-10

**Authors:** Raphaël Scherrer, Colin M. Donihue, Robert Graham Reynolds, Jonathan B. Losos, Anthony J. Geneva

**Affiliations:** ^1^ 15636 Department of Organismic and Evolutionary Biology and Museum of Comparative Zoology Harvard University Cambridge Massachusetts USA; ^2^ 8620 Department of Biology University of North Carolina Asheville Asheville North Carolina USA; ^3^ Present address: 3647 Groningen Institute for Evolutionary Life Sciences University of Groningen Groningen The Netherlands; ^4^ Present address: Department of Biology Washington University St. Louis Missouri USA; ^5^ Present address: Department of Biology Center for Computational and Integrative Biology Rutgers University–Camden Camden New Jersey USA

**Keywords:** adaptation, machine learning, polymorphism, reflectance, sexual signal

## Abstract

Animal signals evolve in an ecological context. Locally adapting animal sexual signals can be especially important for initiating or reinforcing reproductive isolation during the early stages of speciation. Previous studies have demonstrated that dewlap colour in *Anolis* lizards can be highly variable between populations in relation to both biotic and abiotic adaptive drivers at relatively large geographical scales. Here, we investigated differentiation of dewlap colouration among habitat types at a small spatial scale, within multiple islands of the West Indies, to test the hypothesis that similar local adaptive processes occur over smaller spatial scales. We explored variation in dewlap colouration in the most widespread species of anole, *Anolis sagrei*, across three characteristic habitats spanning the Bahamas and the Cayman Islands, namely beach scrub, primary coppice forest and mangrove forest. Using reflectance spectrometry paired with supervised machine learning, we found significant differences in spectral properties of the dewlap between habitats within small islands, sometimes over very short distances. Passive divergence in dewlap phenotype associated with isolation‐by‐distance did not seem to explain our results. On the other hand, these habitat‐specific dewlap differences varied in magnitude and direction across islands, and thus, our primary test for adaptation—parallel responses across islands—was not supported. We suggest that neutral processes or selection could be involved in several ways, including sexual selection. Our results shed new light on the scale at which signal colour polymorphism can be maintained in the presence of gene flow, and the relative role of local adaptation and other processes in driving these patterns of dewlap colour variation across islands.

## Graphical abstract

Animal signals evolve in an ecological context. Here, we show how coloration (as measured by reflectance profiles) of the dewlap in the neotropical lizard *Anolis sagrei* varies among habitats and islands throughout the Bahamas and Cayman Islands.

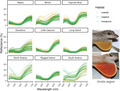

## 1 INTRODUCTION

The staggering diversity of animal communication signals has long been of interest to evolutionary biologists. Animals use chemical, mechanical, electromagnetic and visual signals to communicate in a wide variety of contexts, including competition for mates, species recognition, aposematism and cooperation (Bradbury & Vehrencamp, 2011). A primary evolutionary factor shaping communication signals is the sensory system and behaviour of recipients (the sensory drive hypothesis; Endler, 1992, 1998; Endler & McLellan, 1988). Over the past decades, scientists have established that signals evolve in an ecological context and are dependent on environmental conditions (Endler, 1992, 1993a, 1993b). Just as different habitats may favour different combinations of ecomorphological traits to maximise performance and fitness (Arnold, 1983), they may also shape different forms of a signal, so as to maximise its transmission and detection (e.g. Seehausen, 1997), or reduce its detection by unintended recipients such as predators (Endler, 1984, 1990, 1991; Halfwerk et al., 2014). This selective pressure may drive the local adaptation of communication signals.

One potential barrier to the maintenance of localised signal divergence is the homogenising effect of gene flow. Population genetics theory suggests that gene flow may counteract local adaptation between localities and prevent divergence altogether, especially at small spatial scales, because of the inflow of maladapted alleles or because of the breaking of linkage between coevolving loci (Dieckmann & Doebeli, 1999; Felsenstein, 1976; García‐Ramos & Kirkpatrick, 1997; Hendry et al., 2007a; Lenormand, 2002). This genetic homogenisation has been confirmed empirically in systems such as stick insects (Nosil & Crespi, 2004) and stickleback (Hendry et al., 2007b). Yet, examples of microgeographic adaptation, that is adaptation at smaller scales than the range of dispersal, exist, highlighting the potential of some organisms to respond to selection in the face of gene flow (see Richardson et al., 2014 and references therein). Examples include small scale adaptation in fragmented areas in Australian fruit flies (Willi & Hoffmann, 2012), and local adaptation to predation pressure in North American salamanders (Richardson & Urban, 2013). Therefore, despite evidence that local adaptation may be particularly difficult at small spatial scales where gene flow tends to cause adjoining populations to remain genetically homogeneous, the potential adaptive response of species traits, in particular communication signals, to localised differences in habitats remains relatively unknown (Richardson et al., 2014). Lizards of the neotropical genus *Anolis* (Squamata: Dactyloidae) are an excellent group for studying the eco‐evolutionary dynamics of local adaptation and natural selection (Losos, 2009). A particularly conspicuous trait of anoles is their dewlap, an extensible flap of skin that is typically sexually dimorphic and used as a communication signal in courtship (Driessens et al., 2014, 2015; Sigmund, 1983) and territorial displays (Losos, 1985; Macedonia et al., 2013; Macedonia & Stamps, 1994) as well as in predator deterrence (Leal & Rodríguez‐Robles, 1995, Leal & Rodríguez‐Robles, 1997; Leal & Rodriguez‐Robles, 1997). Dewlap characteristics vary widely among the approximately 400 species of the genus (Nicholson et al., 2007). Interspecific variation in dewlap colouration is implicated in species recognition (Fleishman, 2000; Losos, 1985; Macedonia et al., 2013; Macedonia & Stamps, 1994; Rand & Williams, 1970; Williams, 1969; Williams & Rand, 1977), and this function could have had a role in initiating or reinforcing reproductive isolation during speciation (Geneva et al., 2015; Lambert et al., 2013; Ng et al., 2017).

Within species, studies have shown a link between variation in dewlap colouration and differences in habitats or climatic conditions (Driessens et al., 2017; Leal & Fleishman, 2002, 2004; Macedonia, 2001; Ng et al., 2012, 2013, 2016; Thorpe, 2002; Thorpe & Stenson, 2002; Vanhooydonck et al., 2009). Some studies suggest that those differences may be adaptive and that dewlaps may have evolved to maximise detectability given local light conditions (Fleishman & Persons, 2001; Leal & Fleishman, 2002, 2004). Although this claim is further supported by recent findings that dewlap colours are perceived differently under different levels of shading (Fleishman et al., 2020), other studies found conflicting patterns of between‐habitat variation that did not support the sensory drive hypothesis (Fleishman et al., 2009; Macedonia et al., 2014; Ng et al., 2012).

Previous studies investigating variation in anole dewlaps compared populations at relatively large geographical scales, for example between islands (Driessens et al., 2017; Vanhooydonck et al., 2009) or within large islands such as Puerto Rico (Leal & Fleishman, 2004) or Hispaniola (Ng et al., 2012, 2016). These large scales and marine barriers should reduce gene flow (Lambert et al., 2013; Ng et al., 2017; Ng & Glor, 2011; Richardson et al., 2014). That said, examples do exist of divergence in dewlap colouration at smaller scales or between populations with high degrees of gene flow (Ng et al., 2016; Stapley et al., 2011; Thorpe, 2002; Thorpe & Stenson, 2002).

*Anolis sagrei* is widespread across islands of the West Indies (Reynolds et al., 2020). It has been the subject of numerous studies concerning local adaptation (Kolbe et al., 2012; Losos et al., 1994, 1997, 2001), biological invasion (Kolbe et al., 2008) and sexual selection (Driessens et al., 2014, 2015; Steffen & Guyer, 2014; Tokarz, 2002, 2006; Tokarz et al., 2005) among many other topics. Between‐island variation in the mainly orange‐red colour of its dewlap was shown to be better explained by climatic variables such as annual precipitation and solar radiation (proposed to affect the average vegetation type on each island and among other things, its ambient light environment, Driessens et al., 2017), than by proxies for biotic factors such as sexual selection or predation pressure (Baeckens et al., 2018; Vanhooydonck et al., 2009). How intraisland differences in habitat may contribute to the diversity of dewlap colouration, however, remains unexplored, and may reveal new insights into the scale of local differentiation despite gene flow.

Here, we analysed the colour characteristics of *A*. *sagrei* dewlaps within nine islands in the Bahamas and Cayman Islands. These island systems presently, if not historically, comprise relatively small islands, with no major geographic barriers within islands limiting dispersal for this species. These islands all share three characteristic native West Indian small‐island habitat types—beach scrub bush, closed‐canopy primary coppice forest and mangrove forest—that are often spatially intermingled. These habitats contrast in environmental parameters including vegetation community, light irradiance, humidity and temperature (Howard, 1950; Schoener, 1968). The Cayman Islands and the Bahamas have been colonised independently by *A*. *sagrei* from Cuba (van de Schoot, 2016 unpublished thesis; Reynolds et al., 2020), such that these archipelagos constitute an ideal suite of natural replicates to explore within‐island dewlap diversity across multiple islands.

Our sampling design included sites in close proximity; the median distance between two sites within an island was 8.45 km. Although this species has traditionally been considered territorial, a recent study revealed that they are polygynandrous and that gene flow is not impeded by territorial‐like behaviours exhibited by some males (Kamath & Losos, 2018). Combining reflectance spectrometry and supervised machine learning, we tested for divergence in dewlap phenotype between habitats within islands and between islands across part of the range of *A*. *sagrei*. We predicted that if light conditions in the environment indeed drive colour evolution, dewlaps should be most similar between beach scrub and mangrove forest, which both have high levels of light irradiance, compared to the darker, closed‐canopy coppice forest. If detectability is maximised given the local conditions, we expected darker and more contrasting dewlaps in high irradiance habitats. Finally, if habitat characteristics are strong determinants of dewlap colour variation, similar patterns should be observed across multiple islands (Harvey & Pagel, 1991; Losos, 2011).

## 2 METHODS

### 2.1 Data collection

**FIGURE 1:**
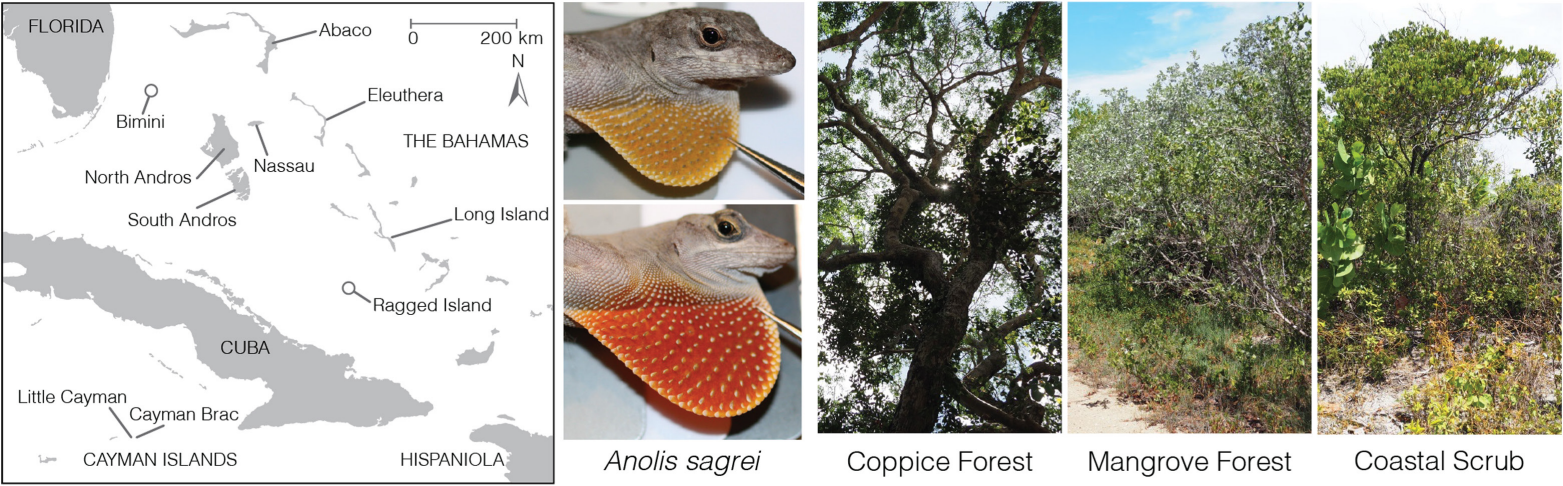
Overview of our study design, including a map of the Bahamas and the Cayman Islands, on which are indicated the nine islands we sampled, two representatives of our study species *Anolis sagrei* with their dewlaps deployed, and the three types of habitats we considered on each island

We sampled 455 male *A*. *sagrei* from seven islands in the Bahamas Archipelago—Abaco, North Andros, South Andros, South Bimini, Eleuthera, Long Island and Ragged Island—and two in the Cayman Islands—Cayman Brac and Little Cayman (Figure 1, S1A). These islands were chosen to span the breadth of the West Indian range of *A*. *sagrei*, because they have highly similar habitat types, and because the *A*. *sagrei* on each island group are derived from ancient and distinct colonisation events from Cuba (i.e. relatively evolutionarily independent, Reynolds et al., 2020). Three habitats were sampled on each island based on characterisations by Howard (1950) and Schoener (1968). Each habitat is clearly distinguishable by its dominant vegetation type—xeric beach scrub (open, relatively dry habitat consisting of low vegetation or isolated trees), primary coppice forest (closed‐canopy forest) and mangrove forest (wet coastal habitat with trees growing in brackish water and high light penetration, although lizards were sampled in dry soil areas). Sample sizes are given in Table S1. Our sampling design enabled us to test for differences between habitats at a coarse and fine geographical scale. The median distance between two localities within an island was 8.45 km (Figure S1B), and 79.3% of all pairwise distances within islands were less than 50 km. Additionally, there are no major barriers to dispersal (such as mountains or grassland) on any of the islands that we sampled.

### 2.2 Reflectance measurements

We measured reflectance between 300 nm and 700 nm wavelength, a range from ultraviolet to red that encompasses the colours visible to most lizards and vertebrates in general (Lazareva et al., 2012). Measurements were taken with an Ocean Optics USB4000 spectrometer, a pulsed Xenon light source (PX‐2, Ocean Optics, Largo, FL, USA) and a reflectance probe protected by a black anodised aluminium sheath. Measurements were taken with a 45‐degree inclination to prevent specular reflection (Endler, 1990). The device was regularly standardised with a Spectralon white standard (Labsphere, North Sutton, NH, USA). Reflectance was measured at the centre of the dewlap. Reflectance curves were smoothed using the R package pavo (Maia et al., 2013) as well as with custom R functions, down to one reflectance value at each nanometre in wavelength from 300 to 700 nm.

### 2.3 Analysis

We tested for detectable differences in dewlap colouration between populations from different habitats across islands by following an analytic pipeline in several steps. First, we used multivariate analyses of variance to assess the relative contributions of islands, habitats and habitat‐by‐island interactions on the partitioning of variation in colour space. Second, and provided that habitat‐by‐island interactions were found, we investigated habitat differences in dewlap colour for each island separately using machine learning classification. Third, for each island where multivariate differences were detected using our machine learning pipeline, we used univariate analyses of variance to decompose the signal among the different dimensions of colour space. Fourth, for each significant between‐habitat variation found in univariate analyses, we used post hoc tests to determine which habitats were responsible for the differences. Last, to obtain insights into the spatial scale of phenotypic variation, for each significant contrast between two habitats detected along a given dimension on a given island, we performed multiple pairwise Wilcoxon tests to assess differences in dewlap colouration among the sites involved in that significant contrast, and recorded the geographical distance between sites that were found significant. In parallel, we tested a possible effect of isolation‐by‐distance, an alternative cause of phenotypic divergence between localities, based on diffusion approximation and dispersal distance, irrespective of habitat types. We did so using a permutation test to assess the significance of the correlation between geographical distances and phenotypic distances among sites within each island.

All analyses in this study were performed in R 3.6.1 (R Core Team, 2019).

### 2.4 Dimensionality reduction

Because neighbouring wavelengths are highly collinear and redundant in reflectance, we reduced the dimensionality of the data using principal component analysis (PCA), as per Cuthill et al. (1999) and Leal & Fleishman (2002). We performed PCA on data from all islands combined, as well as on each island separately and systematically retained the first four principal components (PC), which together always explained more than 88.8% of the variance across islands (Table S2). PCs need not represent the same wavelengths across islands because they are fitted on different datasets. Nevertheless, PC1 was highly collinear with brightness for all islands (Figure S11), whereas the other PCs captured the chromatic variation (i.e. irrespective of brightness) in dewlap colour.

### 2.5 Among‐island variance partitioning

We performed a two‐way nonparametric multivariate analysis of variance (PERMANOVA, Anderson, 2001; R package vegan, Oksanen et al., 2019) to identify differences in colouration between islands, habitats and habitats within islands, using principal components fitted on data from all islands together. We used a nonparametric test because although no multivariate outliers were detected based on the Mahalanobis distance, the assumption of multivariate normality was violated in several habitats on several islands (Henze–Zirkler's test, Henze & Zirkler, 1990; R package MVN, Korkmaz et al., 2014, *p* < 0.05, Table S3).

### 2.6 Within‐island machine learning

We performed a machine learning classification analysis on the first four principal components within each island separately, using random forests (Breiman, 2001). Random forests are a versatile, intuitive and powerful algorithm commonly used in machine learning, using decision trees to predict the labels of particular observations based on their multivariate coordinates. These coordinates, or variables, are passed through a series of successive decision nodes, each examining a given variable of any given observation (James et al., 2013). The prediction for each observation is an aggregate over a large number of decision trees, each tree being trained on a subset of observations sampled with replacement from the data set, and each tree being allowed to examine only a subset of the variables. This allows the random forest to overcome the individual errors of all trees in the predictions it makes.

To detect differences in dewlap colouration between habitats, we measured the success of random forests in reassigning individual lizards to their correct habitat of origin, based solely on their principal component scores. In machine learning, this so‐called cross‐validation procedure is typically done in two steps (James et al., 2013). First, a random forest is trained in recognising features of dewlap colouration most associated with the different habitats, by being presented with multiple observations, making predictions about them and updating its own decision rules based on whether the prediction deviates from the truth. Then, once trained, the patterns that the random forest has learned to recognise are tested by presenting new, previously unseen observations to the random forest, and measuring the proportion of correct predictions. This proportion, or success score, can then be statistically assessed against random guessing using a binomial test.

The cross‐validation procedure requires that the data be split into a training set and a testing set. To remove any bias due to the set that is being sampled for training, it is common practice to use k‐fold cross‐validation (James et al., 2013), where the data are split into *k* random bins and *k* independent machines are trained, each taking one of the bins as a testing set and the rest for training, and where classification success is measured by summing all correct classifications from the *k* machines.

Here, we used a k‐fold cross‐validation procedure with k=5, where each training set consisted of 80% of the data and the machine was tested on the remaining 20%. Each training set was conditioned on containing at least five lizards from each of the three habitats. We also down‐sampled the training set to the sample size of the least represented habitat, to ensure that the different habitats were equally represented. To further remove any bias due to the specific random split into the different bins, we replicated each k‐fold cross‐validation five times. We then averaged the five resulting confusion matrices across replicates, where each confusion matrix shows the number of lizards from each habitat reassigned into each habitat. For each island, we then used the average proportion of correctly reassigned lizards (i.e. the proportion of observations on the diagonal of the average confusion matrix) as an estimate of classification success. This score was tested against random guessing by comparing it to a binomial distribution with number of trials being the number of lizards on that island and success probability 1/3, representing the rate of successful classification by chance when three habitats are involved.

We used the machine learning fitting functions in the R package rminer (Cortez, 2020), which calls random forest routines from the randomForest package (Liaw & Wiener, 2002, implementation from the original random forest algorithm by Breiman, 2001). For each random forest, we optimised the number of trees in the forest and the number of variables examined by each tree using the grid hyperparameter search procedure implemented in rminer, to choose between two numbers of trees (500 or 1,000) and four numbers of principal components examined per tree (1–4), using rminer's ordered holdout validation method with 2/3 of the data used for training.

We validated the results of our analysis by using two other widely used machine learning classification methods: linear discriminant analysis and support vector machines (Cristianini & Shawe‐Taylor, 2000; James et al., 2013), both accessible in rminer (Cortez, 2020).

To know which wavelengths were most used to assign data points to each habitat, we trained another set of random forests, this time directly on reflectance data (taken every 5 nm from 300 to 700 nm) instead of principal components. We recorded the relative importance of each wavelength for each habitat, as measured by the mean decrease in accuracy during wavelength permutation, implemented in the randomForest package (Liaw & Wiener, 2002).

### 2.7 Univariate analyses

For each island where significant differences in dewlap colouration were detected between habitats, we used multiple univariate analyses of variance (ANOVA) to identify possible principal components underlying the observed differences. We constructed our ANOVA models in two steps, as per Zuur (2009). In a first step, we accounted for heterogeneity of variances across groups by systematically comparing the goodness‐of‐fit of an ANOVA model estimated with ordinary least squares (OLS) with that of a model estimated with generalized least squares (GLS), which allowed one estimate of residual variance per habitat (using the R package nlme, Pinheiro & Bates, 2000; Pinheiro et al., 2020). Both models were fitted with restricted maximum likelihood (REML). Goodness‐of‐fit was estimated using Akaike's Information Criterion corrected for small sample sizes (AICc, R package MuMIn, Bartoń, 2019), and the estimation method yielding the lowest AICc was retained. In a second step, we re‐fitted the retained model with maximum likelihood (ML) to test for the effect of habitat type using likelihood ratio tests (LRT) between a model including a habitat term and a null model lacking the habitat term.

We evaluated the normality of the standardised residuals (residuals divided by their standard error, which can differ among habitats in a GLS model) of each fitted ANOVA model using Shapiro–Wilk's test, with *p*‐values adjusted for multiple testing using the Benjamini–Hochberg correction (Benjamini & Hochberg, 1995). In cases where significant deviations from normality were detected ($p_{\mathrm{adj}}<0.05$, Table S4), we performed Kruskal–Wallis's nonparametric test to back up the ANOVA results.

To know which habitat populations were different from which in dewlap colouration, we performed different post hoc multiple comparison tests (all implemented in the PMCMRplus package, Pohlert, 2020), depending on which assumptions were met. In cases where normality and homoscedasticity were met (i.e. OLS‐ANOVA was the best fit), we used Tukey's honest significant difference test. When normality was met but not homoscedasticity (i.e. GLS‐ANOVA was the best fit), we used Dunnett's T3 test. Finally, whenever we used Kruskal–Wallis's test because the ANOVA residuals were not normally distributed, we used Nemenyi's test for post hoc comparisons.

### 2.8 Spatial autocorrelation

We tested for within‐island spatial autocorrelation between the geographical distances among sampling sites and their Euclidean distances in multivariate colour space (mean PC1 to PC4 per site, Table S5), regardless of habitat type. For this, we performed Mantel's test (Legendre & Legendre, 2012, R package vegan; Oksanen et al., 2019) on each island, using 999 permutations and geographical distances computed as geodesic distances from latitude and longitude data (R package geosphere, Hijmans, 2019).

### 2.9 Site differences

In this study, we were interested in the minimum spatial scale at which significant differences between habitats could be detected within islands. We performed multiple pairwise nonparametric Wilcoxon–Mann–Whitney tests (Hollander et al., 2013) to compare dewlap colouration between sites with different habitat types, for each pair of habitats and each variable where significant differences were detected with our analyses of variance. The *p*‐values were adjusted using a Benjamini–Hochberg correction for multiple testing (Benjamini & Hochberg, 1995).

## 3 RESULTS

We tested for variation in *A*. *sagrei* dewlap colouration between populations living in three characteristic habitat types across nine islands that span the West Indian range of the brown anole (beach scrub, primary coppice and mangroves). We found that most of the variation in colouration was partitioned between islands (two‐way PERMANOVA, F(df=8)=45.38, p=0.001, explained variance $R^{2}=41.4$%). Nonetheless, we did find evidence for differences in dewlap colouration between habitat types, and those were mostly island‐specific (habitat‐by‐island interaction term, F(16)=4.78, p=0.001, $R^{2}=8.7$%), with a significant portion of the variation explained by a habitat effect across all islands, but this effect was relatively small (F(2)=4.46, p=0.001, $R^{2}=1$%).

We subsequently tested for differences in dewlap colouration between habitat populations within each island, using within‐island principal component scores (to maximise the variation captured for each island, see Methods). Our within‐island random forest classification analyses revealed detectable differences in dewlap colouration on seven out of the nine islands in our sample: Abaco, Bimini, Cayman Brac, Eleuthera, Little Cayman, Long Island and North Andros. The accuracy of random forest classification exceeded random expectation more often than expected by chance for all these islands (Table 1). Accuracy was as high as 74.8% for Cayman Brac. We obtained similar results using other machine learning approaches such as support vector machines (Table S7) and linear discriminant analysis (Table S8). We describe in details the specific differences detected on each island in Appendix A and focus here on the general patterns emerging from our data.

**TABLE 1:** Random forest classification results

Island	*N*	Score	p
Abaco	86	0.623	<0.0001***
Bimini	57	0.460	0.0194*
Cayman Brac	50	0.748	<0.0001***
Eleuthera	55	0.520	0.0023**
Little Cayman	45	0.676	<0.0001***
Long Island	53	0.611	<0.0001***
North Andros	28	0.693	<0.0001***
Ragged Island	50	0.412	0.1259
South Andros	31	0.419	0.1152

Note

For each island are shown the sample size (*N*) and the proportion of correctly reassigned observations (or success score). *p*‐values were computed using a binomial test and assess the significance of the observed success score relative to the score expected under random guessing.

* p<0.05;

** p<0.01;

*** p<0.001.

Overall, we found significant differences in dewlap colouration between populations that were often in close geographical proximity. On Bimini, notably, we found a significant difference between dewlaps from beach scrub and primary coppice forest, at a distance of a few hundred metres, making this contrast the smallest geographical scale at which differences in colouration were found in our study (Figure S3). We also detected significant differences in dewlap colouration at distances below one kilometre on Abaco (Figure S2G), and at distances between one and ten kilometres on Bimini (Figure S3G), Cayman Brac (Figure S4G), Little Cayman (Figure S6G), Long Island (Figure S7G) and North Andros (Figure S8G).

**FIGURE 2:**
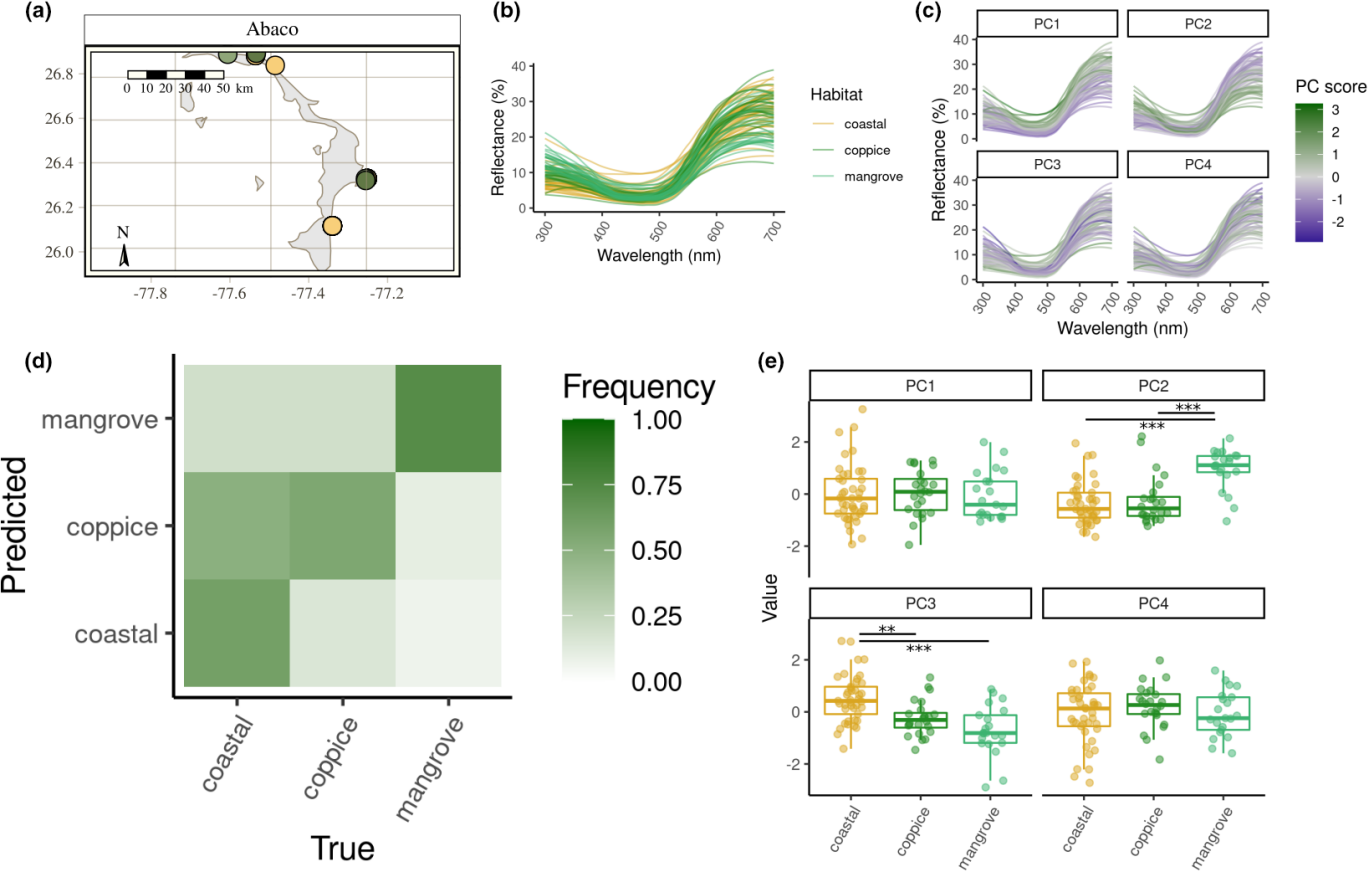
Comparison of dewlap colouration across habitats on Abaco. (a) Map of the island with the sampling sites coloured by habitat. (b) Reflectance profiles of all the dewlaps on the island. (c) How reflectance profiles map onto the within‐island principal components. (d) Confusion matrix showing the proportion of lizards from each (true) habitat reassigned to each (predicted) habitat by the random forests, based on the first four within‐island principal components and averaged across replicates. Each column sums to one. (e) Within‐island principal component scores across habitats. Bars indicate significant contrasts. *p<0.05; **p<0.01; ***p<0.001

We found evidence of spatial autocorrelation in dewlap colouration between the sites within islands for Abaco (Table 2), suggesting that populations from closer sites tend to have more similar dewlaps on this island than expected by chance. Abaco was the island we sampled at the largest scale, with some sites nearly a hundred kilometres away from each other (Figure 2a). That said, some sites were also in close proximity, and significant differences in colouration were detected between habitats sometimes less than a kilometre away (Figure S2G), suggesting that differences in dewlap colouration between distant sites may be partly attributable to isolation‐by‐distance, but this may not necessarily be the case for sites in close proximity. We did not find evidence for spatial autocorrelation on other islands than Abaco (although Eleuthera was nearly significant, Table 2).

**TABLE 2:** Mantel's test of spatial autocorrelation

Island	ρ	p
Abaco	0.439	0.032*
Bimini	−0.725	1.000
Cayman Brac	−0.737	0.833
Eleuthera	0.827	0.058
Little Cayman	−0.042	0.667
Long Island	−0.077	0.583
North Andros	−0.968	1.000
Ragged Island	−0.363	0.708
South Andros	0.963	0.167

Note

For each island are shown the correlation (Pearson's ρ) between the matrix of phenotypic distances between populations from each site and the matrix of geographic distances between sites, where phenotypic distances are Euclidean distances between the mean phenotypes of each site in the multivariate space consisting of the first four within‐island principal components. P‐values assess the significance of the observed correlation against the correlation expected if population means were randomly permuted among sites (999 permutations).

* *p* < 0.05.

A striking feature of our data was inconsistency in between‐habitat differences among islands, in terms of which habitats differ, which dimensions of colouration were involved, and in which direction. For example, while on Cayman Brac the random forests could well distinguish between all three habitats (Figure S4D), on Abaco dewlaps from beach scrub and primary coppice were often mistaken and on Bimini beach scrub dewlaps were more often classified into primary coppice or mangrove than into beach scrub (Figure S3D). In terms of variable importance, for multiple islands the random forests used information in the UV range to discriminate between at least some habitats, particularly on Abaco (Figure S2F), Bimini (Figure S3F), Cayman Brac (Figure S4F), Little Cayman (Figure S6F) and Long Island (Figure S7F), but differences in UV reflectance involved different habitats and were in different directions among these islands (Table 3).

**TABLE 3:** Significance of habitat differences in dewlap colouration, using ANOVA for all islands where significant multivariate differences in dewlap colouration were detected by random forests.

Island	Variable	AICc	ΔAICc	AICw	Model	Log‐lik.	*χ* ^2^	df	*p*
Abaco	PC1	255.81	2.06	0.737	OLS	−121.46	0.14	2	0.9318
Abaco	PC2	225.29	3.98	0.880	OLS	−105.64	31.77	2	<0.0001***
Abaco	PC3	229.85	1.44	0.673	OLS	−108.01	27.04	2	<0.0001***
Abaco	PC4	254.59	0.72	0.589	OLS	−120.82	1.41	2	0.4945
Bimini	PC1	162.92	−0.32	0.540	GLS	−72.43	10.03	2	0.0066**
Bimini	PC2	165.36	3.08	0.824	OLS	−76.52	7.70	2	0.0212*
Bimini	PC3	163.58	3.13	0.827	OLS	−75.58	9.59	2	0.0083**
Bimini	PC4	172.47	2.43	0.771	OLS	−80.27	0.20	2	0.9035
Cayman Brac	PC1	136.64	−4.05	0.884	GLS	−59.29	13.81	2	0.0010**
Cayman Brac	PC2	144.75	3.51	0.853	OLS	−66.24	8.41	2	0.0149*
Cayman Brac	PC3	127.13	2.77	0.800	OLS	−56.86	27.16	2	<0.0001***
Cayman Brac	PC4	147.37	4.33	0.897	OLS	−67.63	5.63	2	0.0600
Eleuthera	PC1	166.33	2.26	0.756	OLS	−77.29	0.49	2	0.7827
Eleuthera	PC2	155.78	−2.38	0.767	GLS	−68.74	12.80	2	0.0017**
Eleuthera	PC3	160.47	−0.22	0.527	GLS	−71.18	5.59	2	0.0613
Eleuthera	PC4	160.61	3.85	0.873	OLS	−74.27	6.54	2	0.0380*
Little Cayman	PC1	130.60	2.50	0.777	OLS	−59.26	8.18	2	0.0167*
Little Cayman	PC2	112.66	−3.61	0.859	GLS	−46.74	29.76	2	<0.0001***
Little Cayman	PC3	118.32	1.41	0.669	OLS	−52.68	21.34	2	<0.0001***
Little Cayman	PC4	135.58	2.53	0.780	OLS	−61.92	2.85	2	0.2410
Long Island	PC1	145.51	3.73	0.866	OLS	−66.41	16.58	2	0.0003***
Long Island	PC2	158.82	−1.29	0.656	GLS	−70.56	1.35	2	0.5103
Long Island	PC3	154.36	3.02	0.819	OLS	−71.10	7.19	2	0.0274*
Long Island	PC4	155.59	0.47	0.558	OLS	−71.75	5.89	2	0.0525
North Andros	PC1	89.00	2.87	0.808	OLS	−39.05	0.35	2	0.8406
North Andros	PC2	74.74	−0.37	0.547	GLS	−27.50	17.24	2	0.0002***
North Andros	PC3	87.62	0.25	0.531	OLS	−38.28	1.89	2	0.3893
North Andros	PC4	73.56	5.39	0.937	OLS	−30.40	17.64	2	0.0001***

Note

Model, best‐fitting model (either OLS or GLS). AICc, corrected AIC score of the best‐fitting model. ΔAICc, difference in AICc between the best‐fitting model and the OLS model. AICcw, AICc weight. Log‐lik., log‐likelihood. $\chi^{2}$, likelihood ratio. df, degrees of freedom.

* *p* < 0.05;

** *p* < 0.01;

*** *p* < 0.001.

## 4 DISCUSSION

Two main insights follow from our results. First, we detected significant differences in dewlap colouration between habitats within seven out of the nine islands investigated, suggesting a putatively high potential for local differentiation of dewlap colouration in *Anolis sagrei*. Second, we found differences in colouration along different dimensions of colour space, and in different directions, on different islands.

Detectable differences in dewlap colour between populations are surprising, as habitats were often in close geographical proximity to each other (as close as a few hundred metres on Bimini and most of the time within ten kilometres), and we would have expected gene flow to cause a more homogeneous distribution of colour phenotypes within islands. Although little is known about the cruising range of individuals from our study populations (but see Kamath & Losos, 2018b; Steinberg & Leal, 2017 for other systems), *A*. *sagrei* are polygynandrous (both males and females mate with multiple mates, Kamath & Losos, 2017, 2018a, 2018b), thus offering opportunity for gene flow, especially given that lizards were distributed continuously and at high densities within the islands we sampled. Consistent with that, although populations from different islands were monophyletic, individuals within islands were not monophyletic with respect to habitat based on mitochondrial haplotypes (van de Schoot, 2016 unpublished thesis).

Several scenarios could account for these findings. One explanation is an adaptive one: populations living in different habitats could be phenotypically adapted to their local environmental conditions. Given that the brightly coloured dewlap of *A*. *sagrei* is used as a communication signal, its colour may be a target for selection if the transmission or perception of the signal differs from one habitat to another, for example because of differences in ambient light, according to the sensory drive hypothesis (Endler, 1992, 1998; Endler & McLellan, 1988). The sensory drive hypothesis has been tested multiple times for dewlap colouration in *Anolis* lizards, with mixed results. Some authors found support for it (Leal & Fleishman, 2002, 2004), whereas others found differences in dewlap colouration between habitats inconsistent with the sensory drive hypothesis (Fleishman et al., 2009; Ng et al., 2012).

If our results were an example of sensory drive, we would have expected to see consistent differences between populations from different habitats across islands, given the apparent environmental consistency of each of the three habitat types across the islands we sampled. In particular, we would have expected divergence in line with increased detectability given local light conditions, such as the high contrasts with background vegetation found in the UV range in Leal and Fleishman (2002), and Leal and Fleishman (2004). We might also have expected mangrove and beach scrub lizards, both inhabiting areas with high light penetration, to have more similar dewlaps, and to differ significantly from lizards from the coppice habitat, where irradiance is low. Instead, we found inconsistencies in the way dewlap colour differed between habitats across islands. Although short wavelengths (UV reflectance) were often involved in colour differences, they were not involved on all islands where significant differences were detected. On some islands, other or additional variables differed, such as brightness, red reflectance or the reflectance at the ends of the spectrum visible to *Anolis* lizards (UV and red, Lazareva et al., 2012) relative to intermediate wavelengths (blue‐to‐yellow). Similar portions of the spectrum were sometimes involved in opposite directions on different islands, such as on Abaco and Cayman Brac, where mangrove lizards had a higher UV reflectance than beach scrub lizards on the former, but a lower UV reflectance on the latter. Overall, the observed heterogeneity of divergence patterns across islands provides no support to a sensory drive explanation.

It is presently not known if the reported differences in colouration have a genetic basis. Yet, we find it unlikely that these differences arose through phenotypic plasticity, as although the carotenoids that partly make up the red and orange colours of anole dewlaps must be found in the diet (Goodwin, 1984; Hill et al., 2002; Hill & McGraw, 2006), studies testing the effect of carotenoid deprivation (Ng et al., 2013; Steffen et al., 2010) and heritability (Cox et al., 2017) of dewlap colouration in *A*. *sagrei* and *A*. *distichus* (another species with a carotenoid‐based dewlap), found little support for phenotypic and developmental plasticity in dewlap colouration. One exception is a study demonstrating that lizards heavily parasitised by skin mites had duller dewlaps (Cook et al., 2013), but we found no sign of that in our study.

Genetic drift could contribute to some of the observed variation. Indeed, although only Abaco showed significant patterns consistent with isolation‐by‐distance, which may emerge under limited dispersal and drift (Kimura & Weiss, 1964; Slatkin, 1987; Wright, 1943), there may have been too few sites on most islands to conclusively detect it, and even then, the absence of detectable isolation‐by‐distance may not necessarily constitute evidence for the absence of drift. Besides, spatial autocorrelation was the strongest on islands sampled at the largest scales (e.g. some sites on Abaco were nearly 100 km apart, and Eleuthera—the second strongest signal, albeit nonsignificant—had sites more than 30 km apart), such that it is possible that neutral processes and/or dispersal limitations might contribute to shaping variation over long distances. That said, many significant differences were found between habitats in close proximity, contrary to what would be expected under isolation‐by‐distance, including on islands where spatial autocorrelation was detected. Moreover, *A*. *sagrei* was distributed across the islands continuously, usually at relatively high population densities, rather than in small and isolated populations particularly prone to drift. Together with the fact that isolation‐by‐distance may not necessarily only emerge from drift (e.g. if there is a spatial environmental gradient), this indicates that genetic drift may have limited potential to explain the differences observed between habitats, at least at a local scale.

In this study, we found larger differences among than within islands, a pattern already reported and linked to climatic conditions (Driessens et al., 2017) and to densities of predators and of anole congeners (Baeckens et al., 2018; Vanhooydonck et al., 2009). Differences among habitats within islands, however, are still difficult to account for. Remaining hypotheses may include, for example, runaway sexual selection (i.e. arbitrary preferences of females for some colours over others, Andersson, 1994) operating in different directions across islands, but no evidence so far suggests that dewlap is a target of mate choice in anoles (Lailvaux & Irschick, 2006; Nicholson et al., 2007; Tokarz, 2002; Tokarz et al., 2005). Another hypothesis is that the different genetic constitutions of different islands, perhaps resulting from founder effects (the islands have been colonised independently, van de Schoot, 2016 unpubl.; Driessens et al., 2017, Reynolds et al., 2020), may have predisposed populations to adapt differently to similarly selective circumstances. Either way, new data would be needed to test these hypotheses. Visual modelling, for instance, would be a valuable follow‐up analysis as it would indicate whether the differences we detected may be detectable by the organisms themselves (as has been done in *Anolis* in Leal & Fleishman, 2004, Fleishman et al., 2020), but such approach requires irradiance profiles from the habitats in the field, which we presently do not have.

Last, some patterns may arise owing to factors we did not measure. For one, signalling can be a multifaceted behaviour, and although we focused on dewlap colour here, other potentially important elements of behavioural interactions include dewlap size, dewlap patterning, display activity as well as behaviours associated with dewlap extensions such as headbobs and push‐ups (Driessens et al., 2014, 2015; Lailvaux et al., 2015; Vanhooydonck et al., 2005). These have been linked in various ways (and not always consistently across studies) not only with environmental variables but also with proxies for sexual selection, predation and species recognition (Baeckens et al., 2018; Driessens et al., 2017; Lailvaux & Irschick, 2007; Vanhooydonck et al., 2005, 2009), which do vary among islands but may also vary within islands, possibly in interaction with dewlap colour. Besides, although the three habitats were clearly recognisable and consistent across islands to the human eye (Figure 2), we did not precisely quantify irradiance within each habitat and did not consider any more subtle environmental differences there might have been for a given habitat among islands.

Altogether, our results show that dewlap colour of *A*. *sagrei* commonly varies between habitat types, even in close geographical proximity, within islands of the West Indies. However, colouration differs in different ways across similar habitats from one island to another. We discussed several nonmutually exclusive mechanisms that could explain these observations. Nevertheless, heterogeneous patterns of divergence across islands do not support an adaptive sensory drive scenario, and we propose that within‐island dewlap colour variation may be underlain by a more subtle mosaic of factors.

## CONFLICT OF INTERESTS

The authors declare that they have no conflict of interest.

### PEER REVIEW

The peer review history for this article is available at https://publons.com/publon/10.1111/jeb.14002.

### OPEN RESEARCH BADGES

This article has been awarded Open Data and Open Materials Badges. All materials and data are publicly accessible via the Open Science Framework at https://doi.org/10.5061/dryad.bcc2fqzf4.

## Supporting information

Fig S2‐S10

Appendix S1

## Data Availability

The data that support the findings of this study are openly available in Dryad at https://doi.org/10.5061/dryad.bcc2fqzf4.

## APPENDIX A

Here, we describe more precisely the patterns identified on each island.

On Abaco, dewlaps from the mangrove habitat were the best discriminated, whereas dewlaps from the beach scrub habitat were often mistaken for dewlaps from the coppice habitat (Figure 2d). Importance analysis revealed that beach scrub and mangrove lizards mostly differed in reflectance in the ultraviolet (UV) end of the spectrum (below 400 nm, Figure S2F), where mangrove dewlaps had higher UV reflectance relative to beach scrub lizards, and coppice lizards had an intermediate UV reflectance between the two other habitats (Figure 2b). Consistent with this, our analyses of variance detected significantly higher PC2 scores in mangrove lizards than in the two other habitats (Figure 2e, Table 3), representing a higher UV reflectance relative to red (Figure 2c). Beach scrub lizards also scored higher on PC3 (Figure 2e, Table 3), indicating less curvature of the reflectance profile and relatively higher reflectance at intermediate wavelengths (blue‐to‐yellow) than at the ends of the range (Figure 2c). Differences were detected between sites both at large (~100 km) and short (<1 km) distances (Figure S2G). Abaco was the only island where we detected significant spatial autocorrelation (Table 2), that is, sites that were closer geographically tended to have populations of lizards with more similar dewlap colours.

On Bimini, the random forests mostly correctly classified lizards from the coppice and mangrove habitats while often misclassifying lizards from the beach scrub habitat (Figure S3D). Relatively flat importance profiles for beach scrub lizards suggested that brightness was used instead of a particular wavelength to identify some of the beach scrub dewlaps (Figure S3F). Indeed, some beach scrub dewlaps were substantially brighter than the rest (Figure S3B,C), a pattern that was captured by our analysis of variance along PC1 (i.e. brightness, Figure S3C,E, Table 3). Coppice dewlaps had significantly higher PC2 scores than mangrove dewlaps (Figure S3E), suggesting a higher curvature (higher UV and red reflectance than more intermediate wavelengths, Figure S3C). For these two habitats, the random forests were most sensitive to UV reflectance (Figure S3F). Beach scrub dewlaps had higher PC3 scores than coppice dewlaps but it was not clear what properties of spectral shape this principal component mapped onto (Figure S3C). On this island, the beach scrub and coppice habitats were separated by a few hundred metres, making this contrast the smallest geographical scale at which differences in colouration were found in our study (Figure S3G).

On Cayman Brac, all three habitats could be well discriminated against each other (Figure S4D), with UV reflectance appearing to be an important variable differentiating beach scrub and mangrove dewlaps (Figure S4F). By contrast, coppice dewlaps had a relatively flat importance profile, suggesting that brightness made them more distinct rather than any particular wavelength (Figure S4F). Consistent with this, coppice dewlaps were significantly different from all other dewlaps along PC1 (Figure S4E, Table 3). At a distance between 2 and 3 km (Figure S4G), dewlaps in the beach scrub habitat reflected more red light (as represented by PC2, Figure S4C, E) and more UV (as represented by PC3, along which coppice dewlaps were intermediate, Figure S4C,E) than in the mangrove habitat.

On Eleuthera, although random forests detected between‐habitat differences in dewlap colour, other approaches did not (Tables S7 and S8), suggesting that the differences may be small. On Eleuthera, beach scrub and mangrove dewlaps were guessed relatively correctly, but coppice dewlaps were more often mistaken for mangrove dewlaps (Figure S5D). Mangrove dewlaps had lower PC2 scores than beach scrub dewlaps (Figure S5E, indicating higher UV relative to red, Figure S5C), and higher PC4 scores than coppice dewlaps (Figure S5E, suggesting more reflectance profiles with more curvature, Figure S5C). Random forests did not seem to consistently capture the wavelengths responsible for these differences (Figure S5F).

Little Cayman was characterised by a better discrimination of mangrove lizards from the rest than between beach scrub and coppice lizards even though all habitats were relatively well discriminated (Figure S6D). Mangrove dewlaps were most distinct with respect to their reflectance in short wavelengths (Figure S6F), with significantly lower UV reflectance (as represented by PC2, Figure S6C,E, Table 3). Beach scrub lizards were characterised by brighter dewlaps than coppice lizards (PC1), and also more convex curves, that is slightly higher UV and red reflectance (as represented by higher PC3 scores), than lizards from the other two habitats (Figure S6C,E, Table 3).

On Long Island, the three habitats were relatively well discriminated (Figure S7D). Importance profiles indicated that short wavelengths were used to discriminate between beach scrub and mangrove lizards (Figure S7F). Beach scrub lizards had more curved reflectance profiles than in the mangrove, with higher levels of UV and red reflectance relative to intermediate wavelengths (PC3, Figure S7C,E, Table 3). Coppice lizards were significantly darker than mangrove and beach scrub lizards (PC1, Figure S7C,E, Table 3).

On North Andros, beach scrub and coppice dewlaps could be discriminated better against each other than with mangrove dewlaps (Figure S8D), with importance profiles supporting UV reflectance as a predictor of coppice lizards (Figure S8F). Coppice lizards had less curved reflectance profiles than beach scrub and mangrove lizards (PC2), and beach scrub dewlaps had the lowest scores on PC4, which was difficult to interpret (Figure S8C,E, Table 3).

Classification success was not significantly better than expected by chance on Ragged Island and South Andros (Table 1, Tables S7 and S8) where nearly no habitat could be differentiated from any other based on reflectance.
