# Supplementary figures and images for: Dewlap colour variation in Anolis sagrei is maintained among habitats within islands of the West Indies
Source: J Evol Biol. 2022 May 10;35(5):680–92. doi: 10.1111/jeb.14002 (PMC9321103; doi:10.1111/jeb.14002)

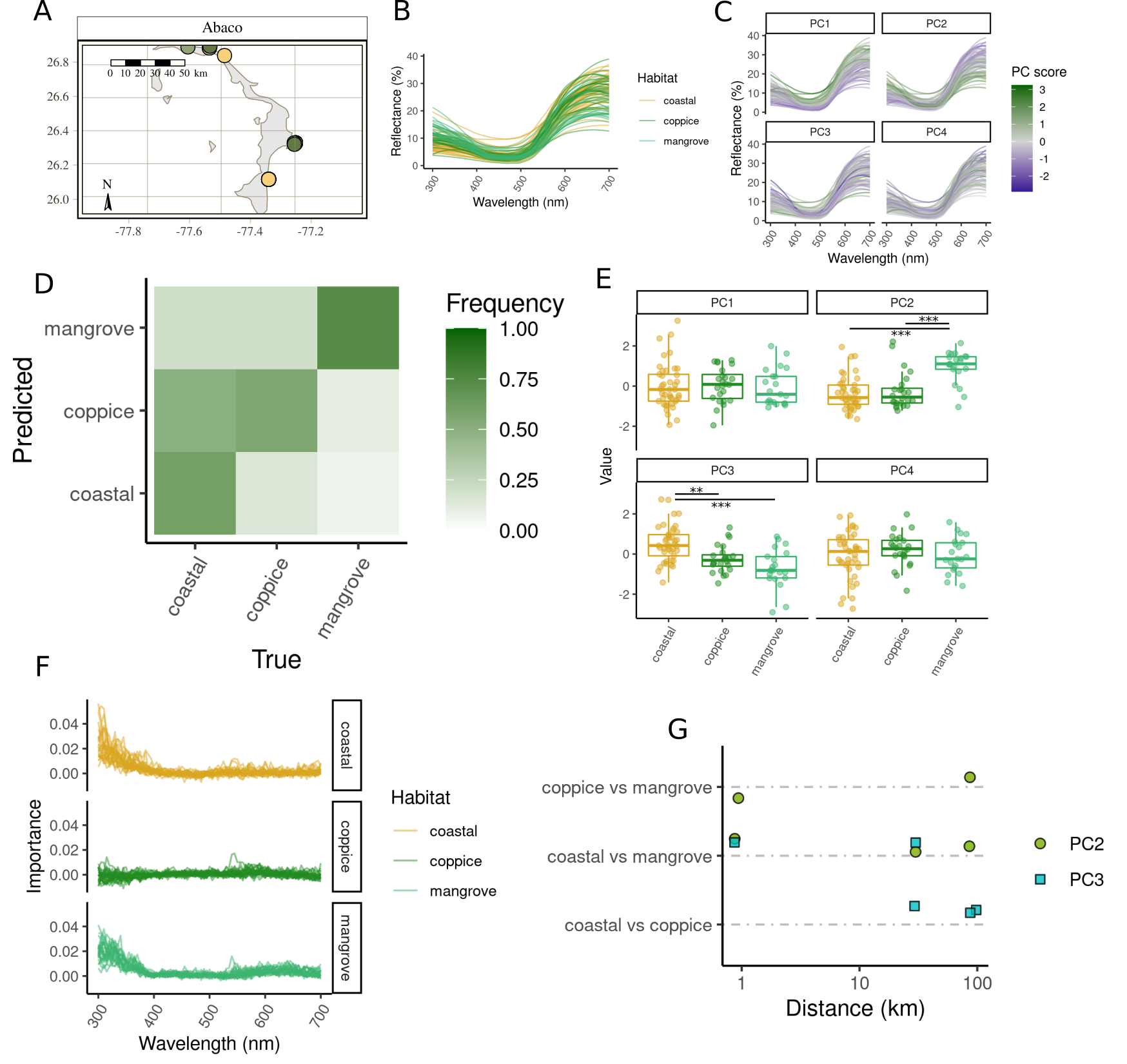

Supplement: Supplementary file 1 — Fig S2‐S10 [file JEB-35-680-s002.zip › jeb14002-sup-0001-Abaco_supplement.png]

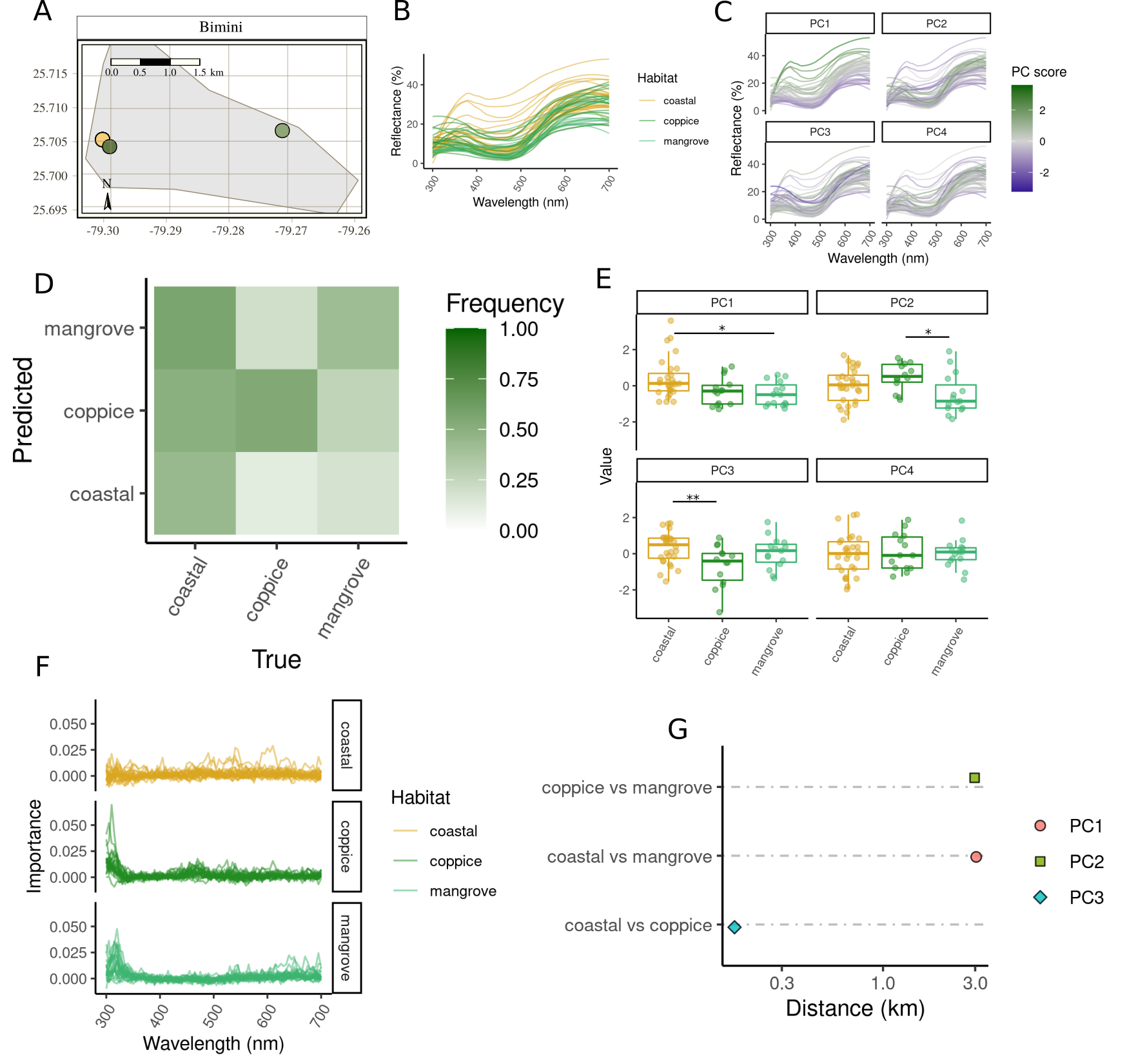

Supplement: Supplementary file 1 — Fig S2‐S10 [file JEB-35-680-s002.zip › jeb14002-sup-0002-Bimini_supplement.png]

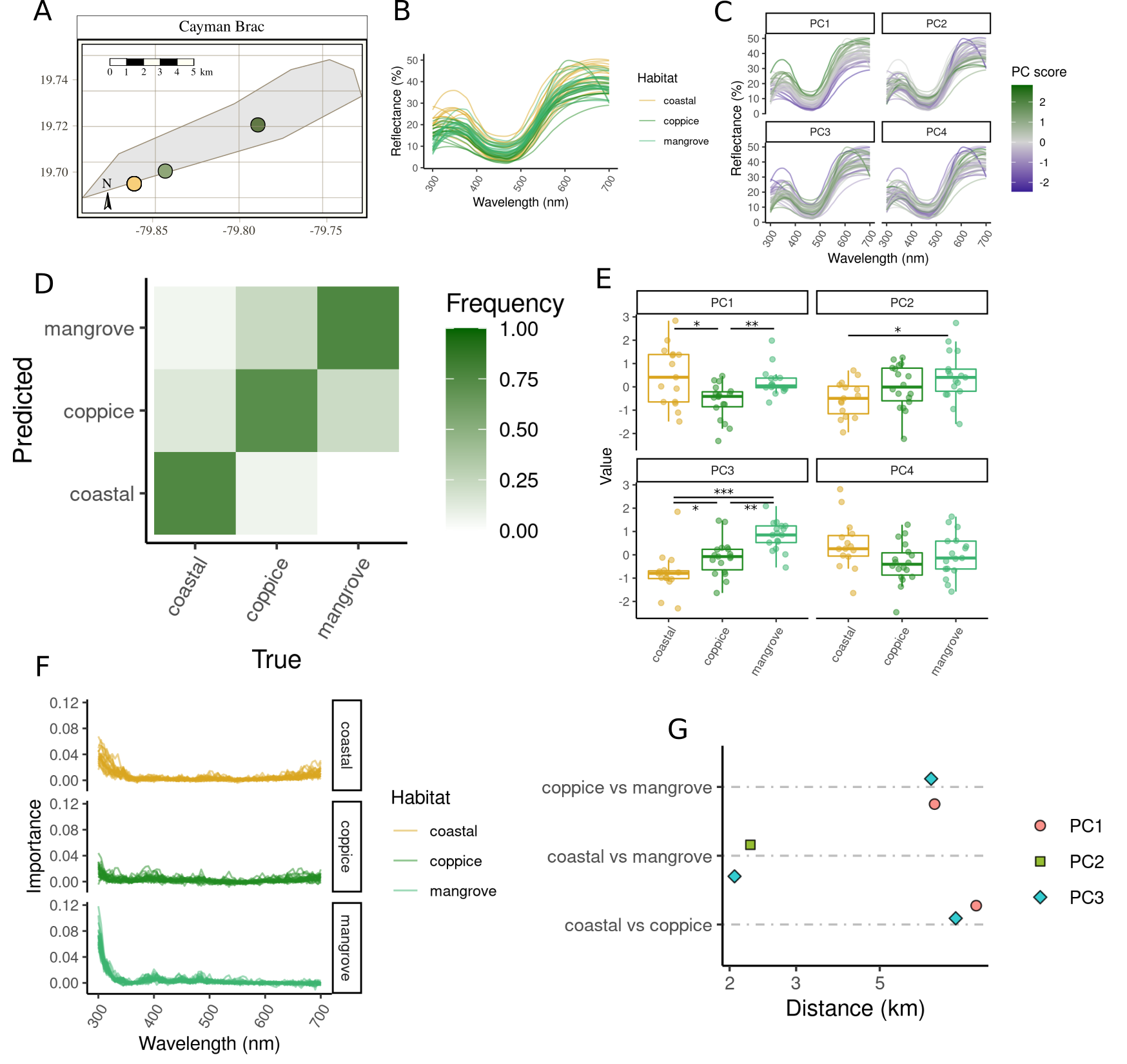

Supplement: Supplementary file 1 — Fig S2‐S10 [file JEB-35-680-s002.zip › jeb14002-sup-0003-CaymanBrac_supplement.png]

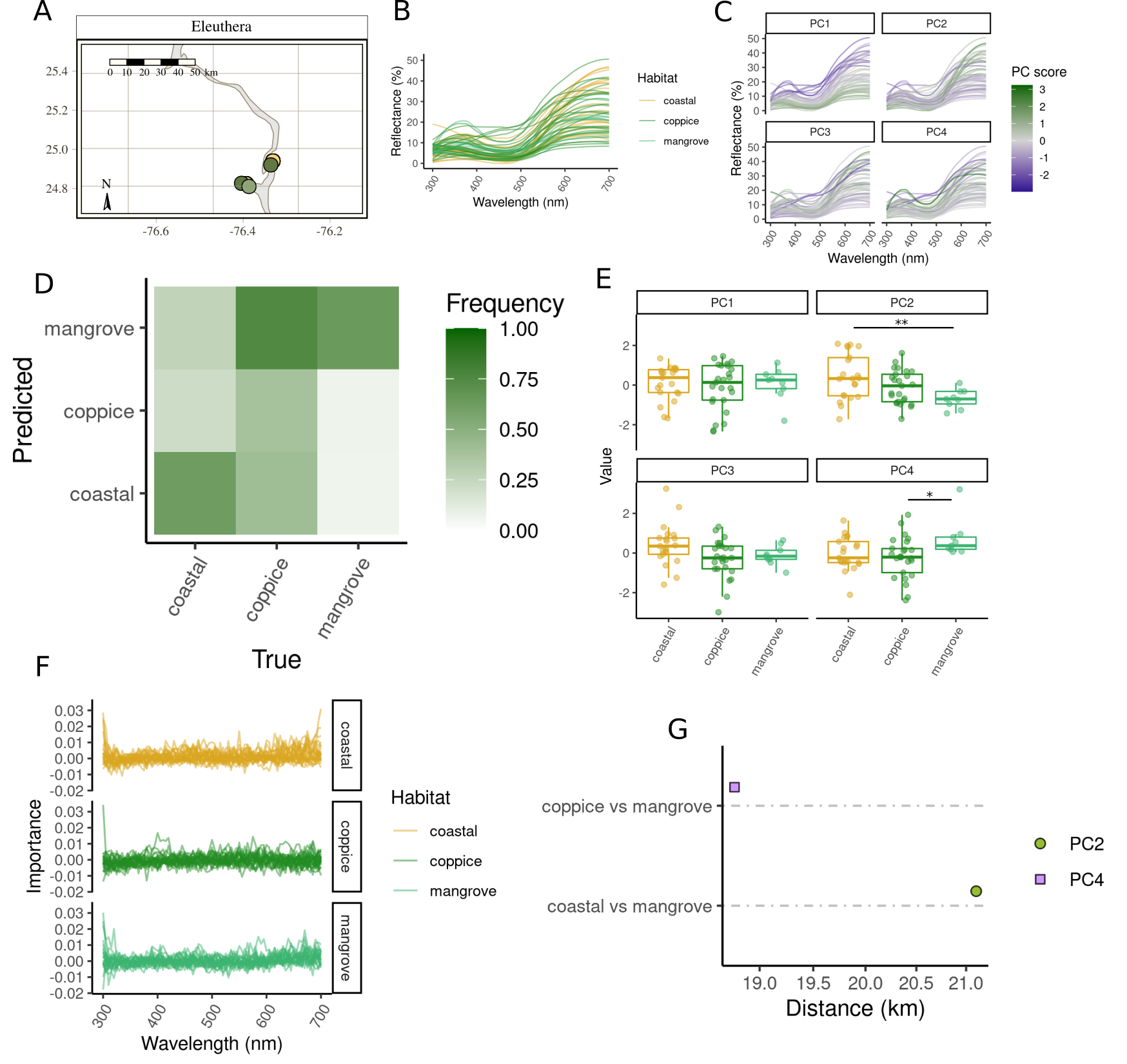

Supplement: Supplementary file 1 — Fig S2‐S10 [file JEB-35-680-s002.zip › jeb14002-sup-0004-Eleuthera_supplement.png]

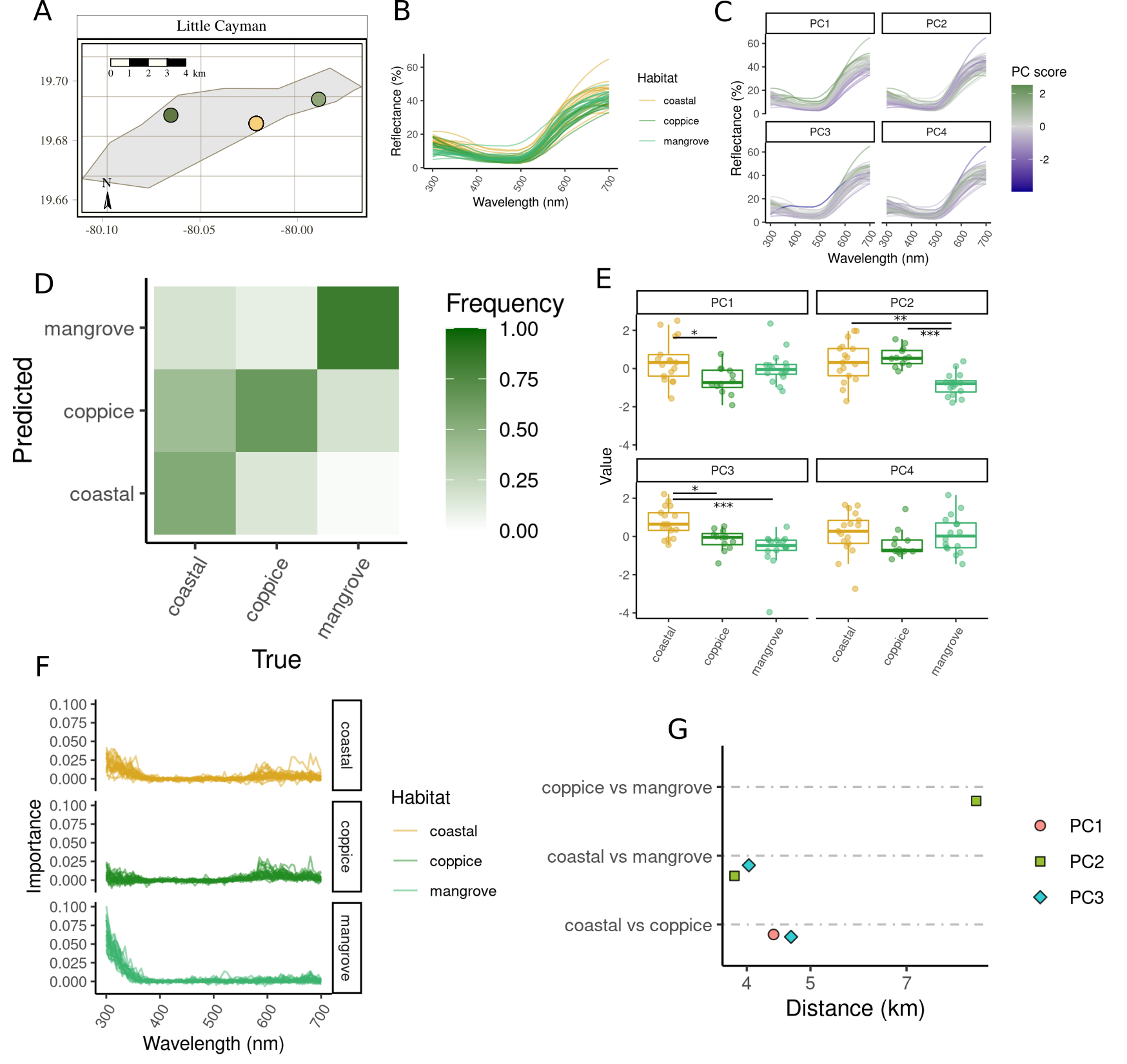

Supplement: Supplementary file 1 — Fig S2‐S10 [file JEB-35-680-s002.zip › jeb14002-sup-0005-LittleCayman_supplement.png]

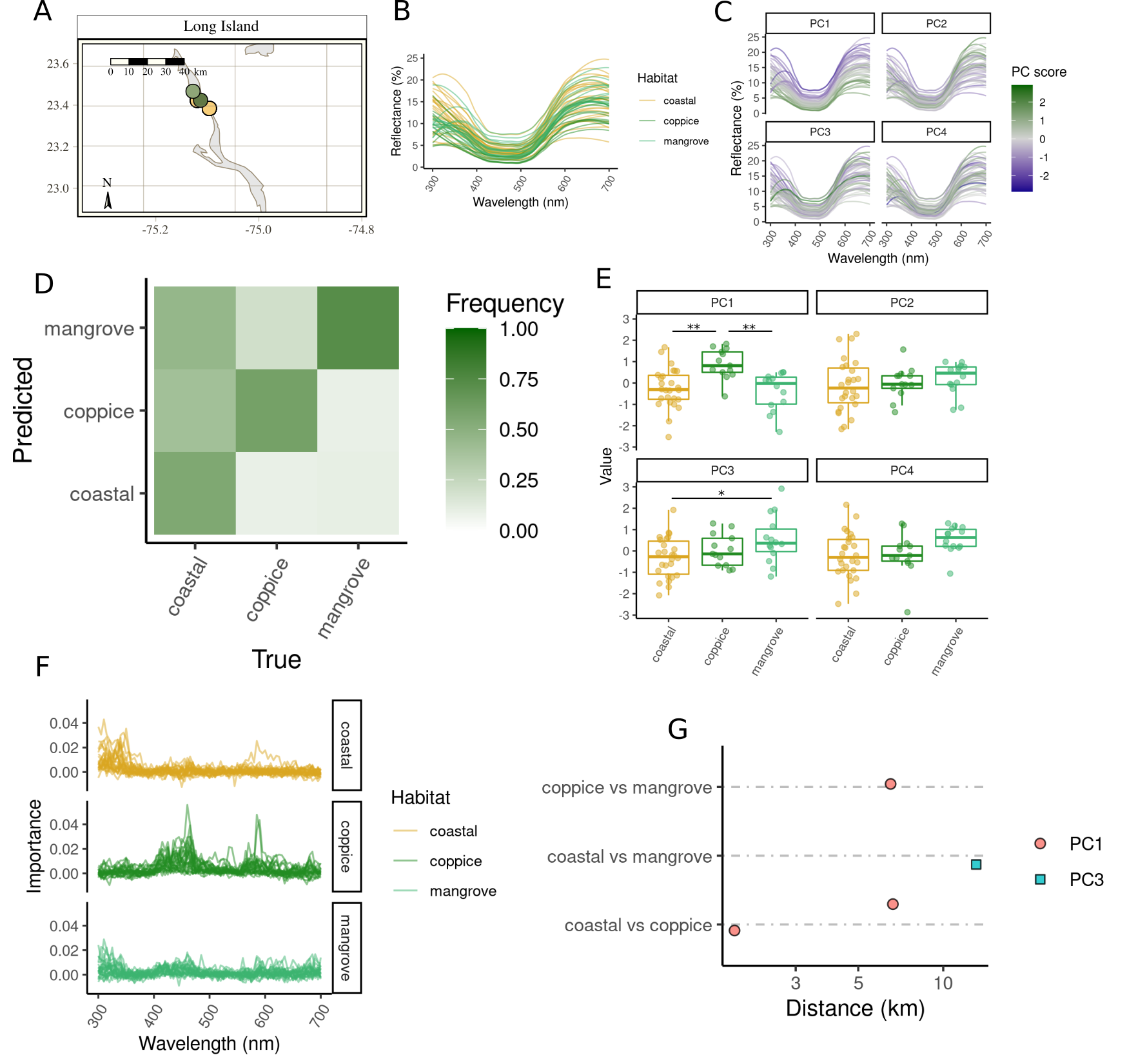

Supplement: Supplementary file 1 — Fig S2‐S10 [file JEB-35-680-s002.zip › jeb14002-sup-0006-LongIsland_supplement.png]

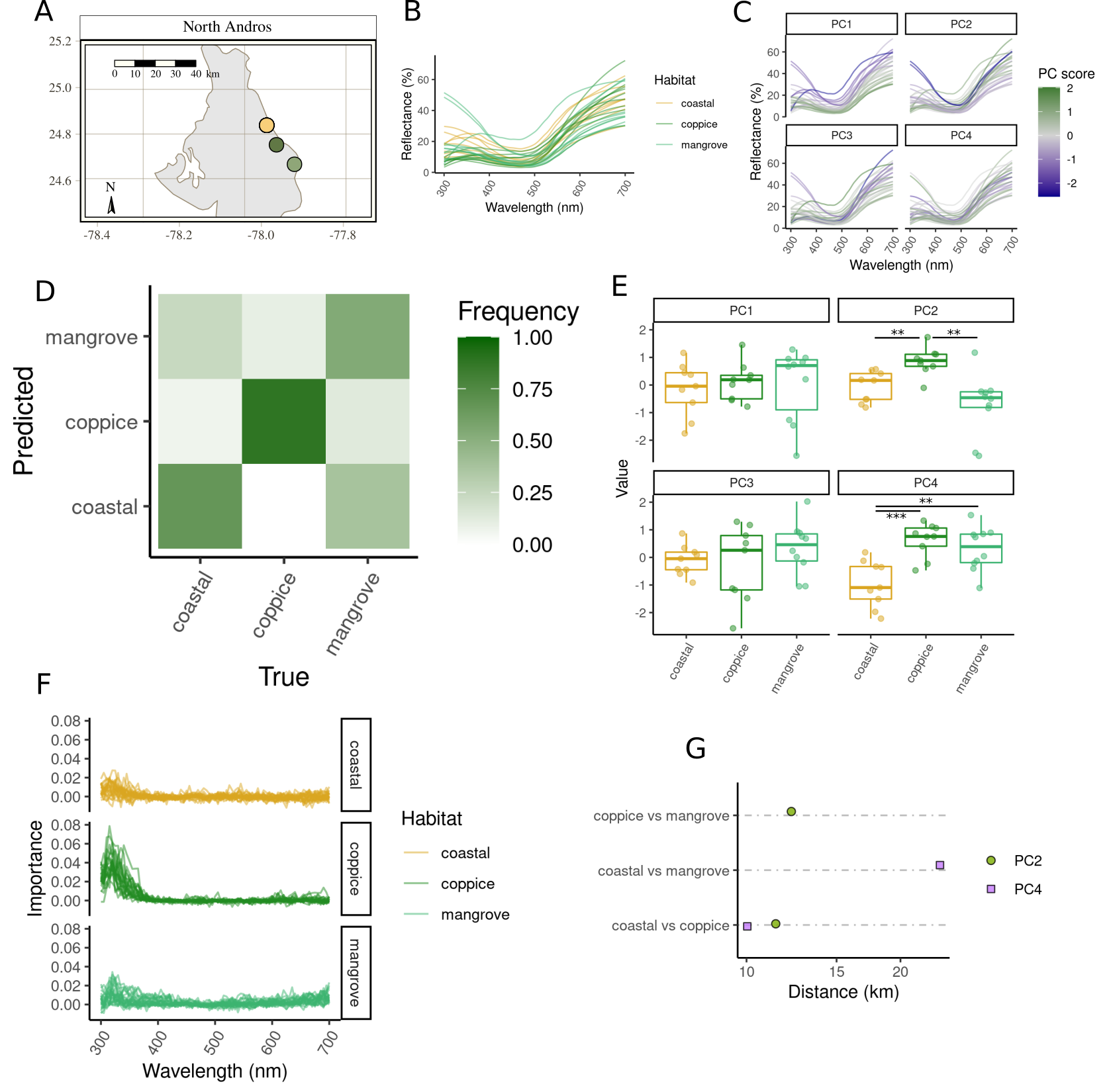

Supplement: Supplementary file 1 — Fig S2‐S10 [file JEB-35-680-s002.zip › jeb14002-sup-0007-NorthAndros_supplement.png]

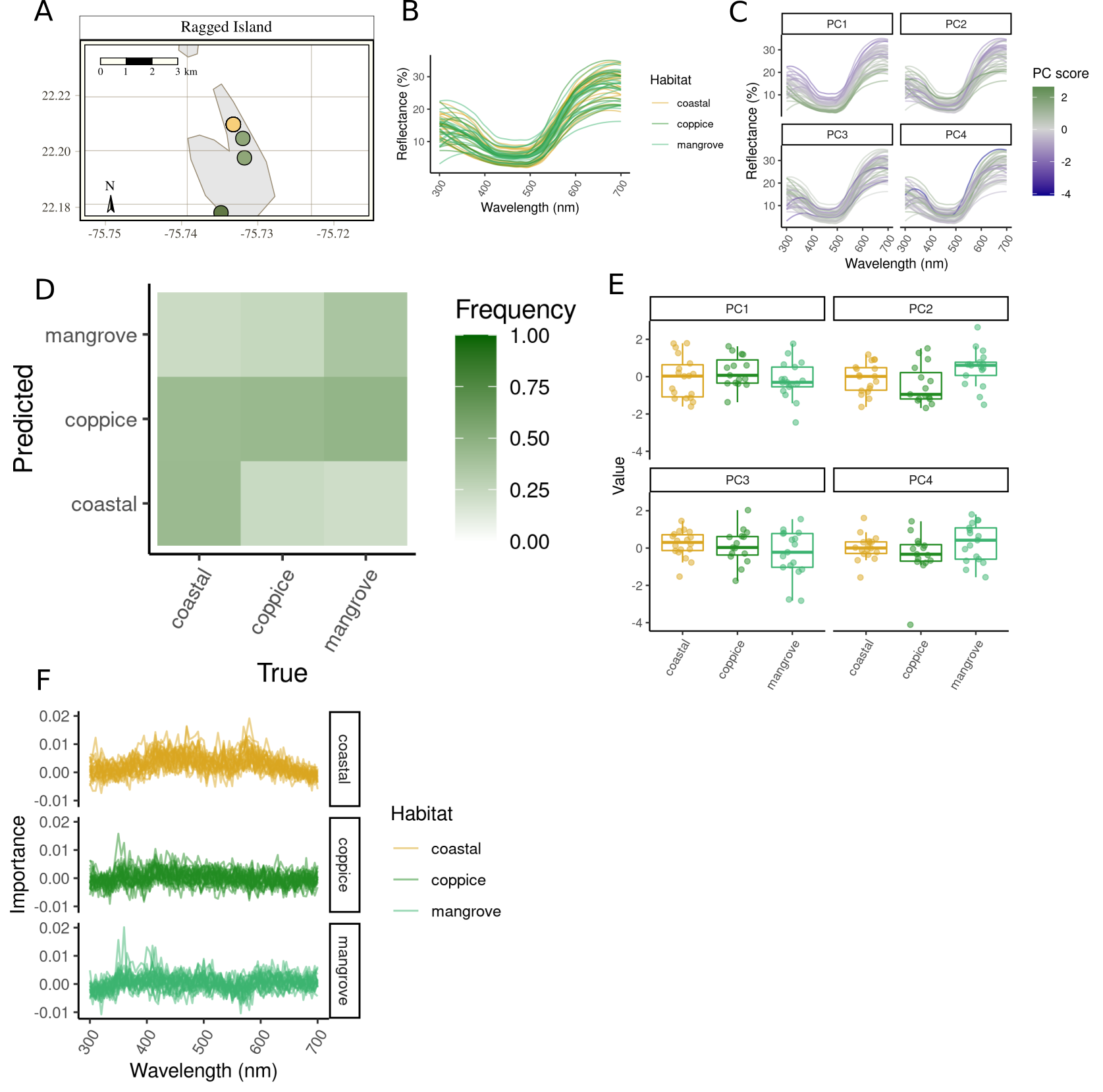

Supplement: Supplementary file 1 — Fig S2‐S10 [file JEB-35-680-s002.zip › jeb14002-sup-0008-RaggedIsland_supplement.png]

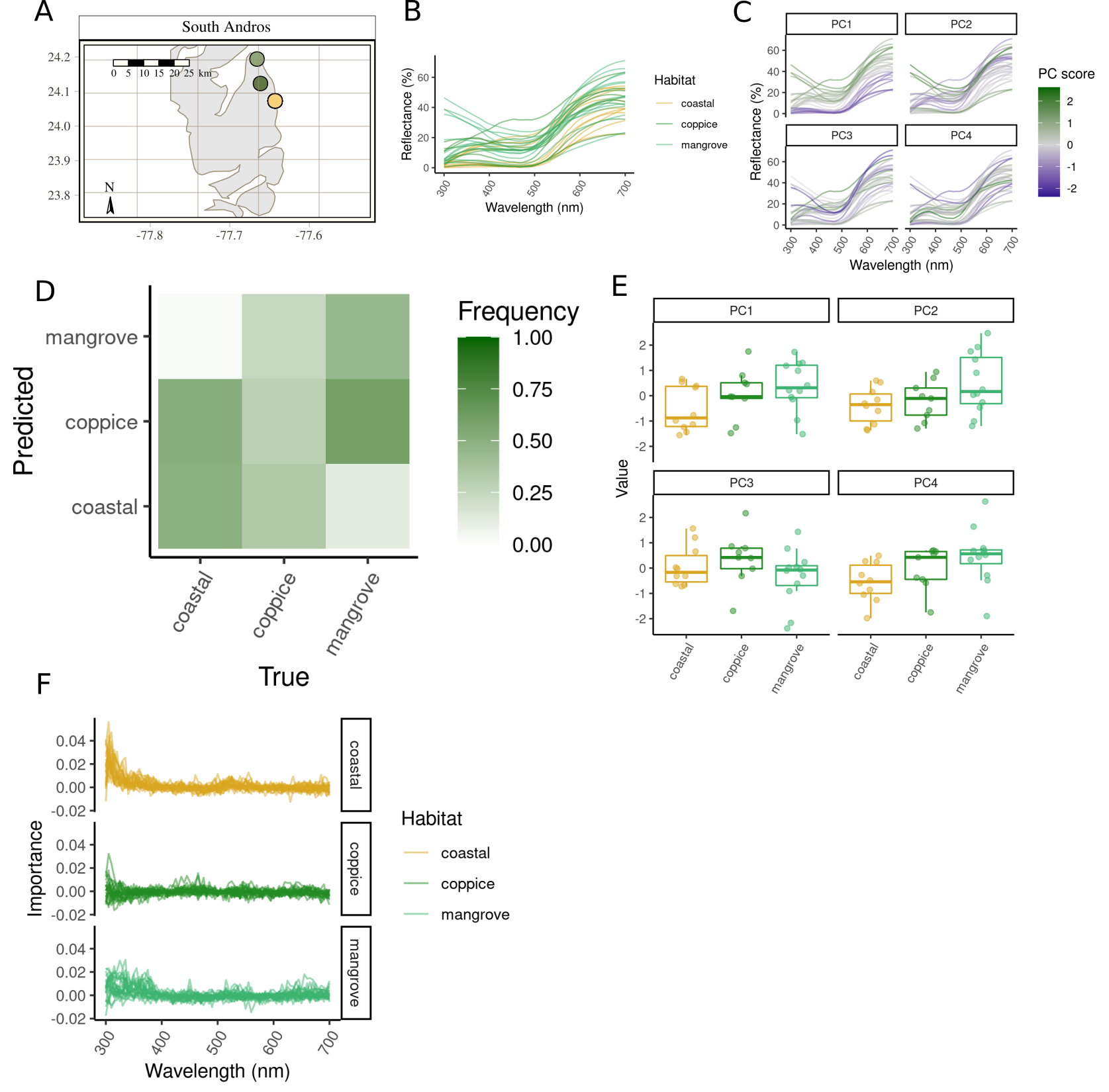

Supplement: Supplementary file 1 — Fig S2‐S10 [file JEB-35-680-s002.zip › jeb14002-sup-0009-SouthAndros_supplement.png]
