# Supplementary material for: Dewlap colour variation in Anolis sagrei is maintained among habitats within islands of the West Indies
Source: J Evol Biol. 2022 May 10;35(5):680–92. doi: 10.1111/jeb.14002 (PMC9321103; doi:10.1111/jeb.14002)
Supplement: Supplementary file 2 — Appendix S1 [file JEB-35-680-s001.pdf]

Supplementary Figures

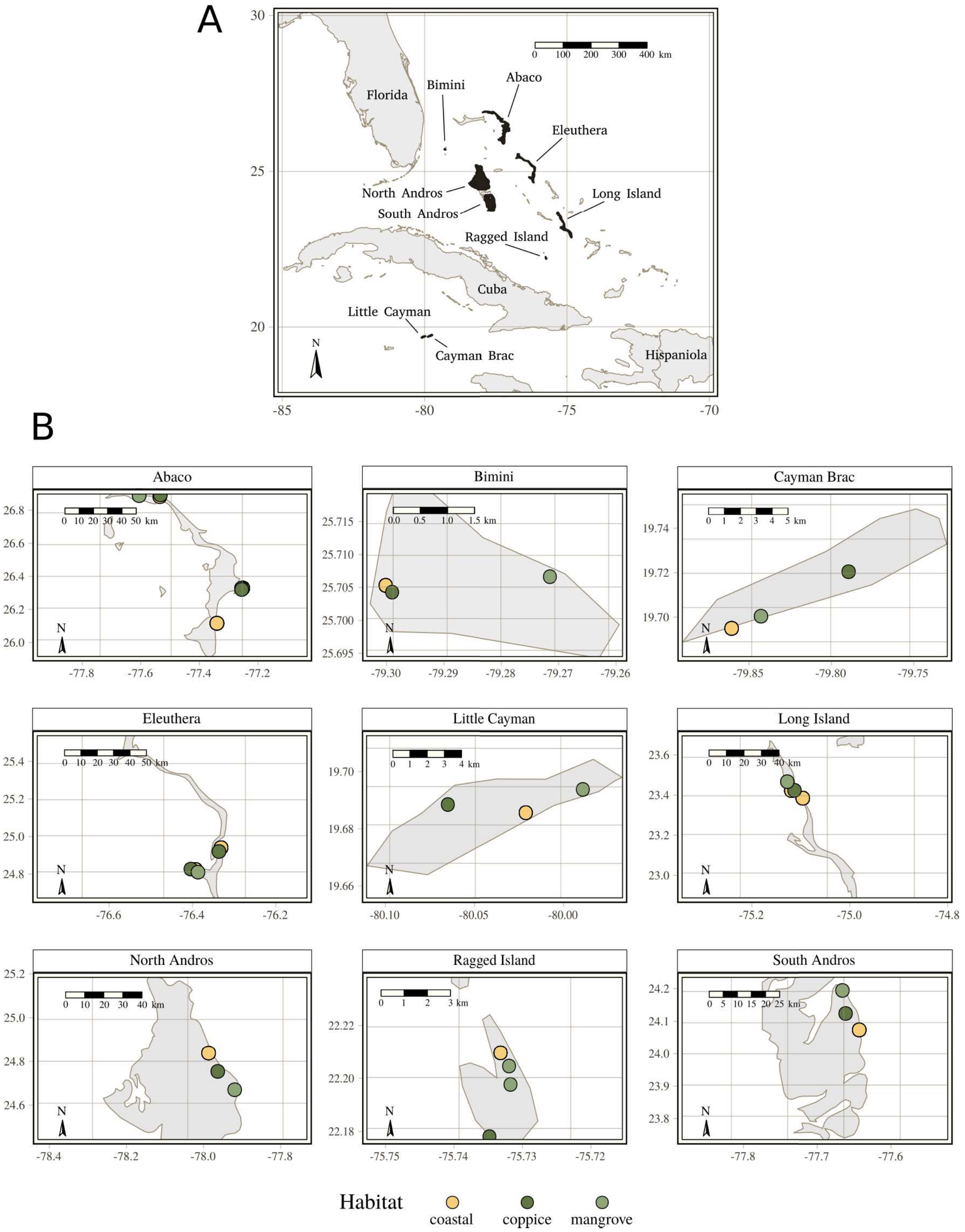

Figure S1: Maps of the islands. (A) Map of the West Indies with sampled islands highlighted in black. (B) Sampling sites within islands colored after their respective habitat types.

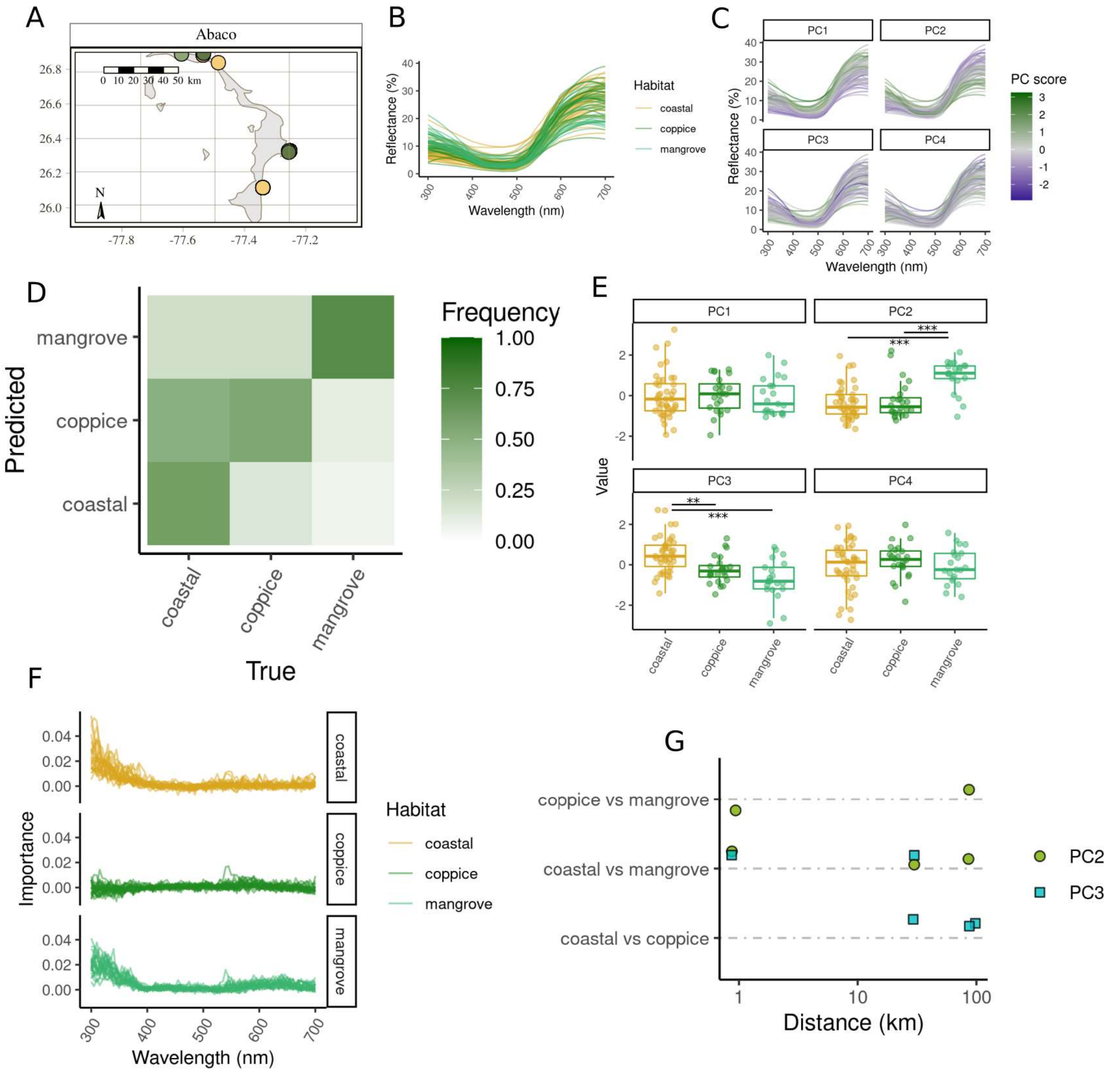

Figure S2: Comparison of dewlap coloration across habitats on Abaco, with extended results. (A–E) Legend as per Figure 2. (F) One-dimensional sensitivity analysis showing the relative importance (mean decrease in accuracy) of the various wavelengths in random forest classification of the whole spectrum. (G) Geographical distance between sites where significant differences were detected in within-island principal component scores (Wilcoxon test, Benjamini-Hochberg correction, $P < 0.05$), including only pairs of sites whose habitats were involved in between-habitat dewlap differences.

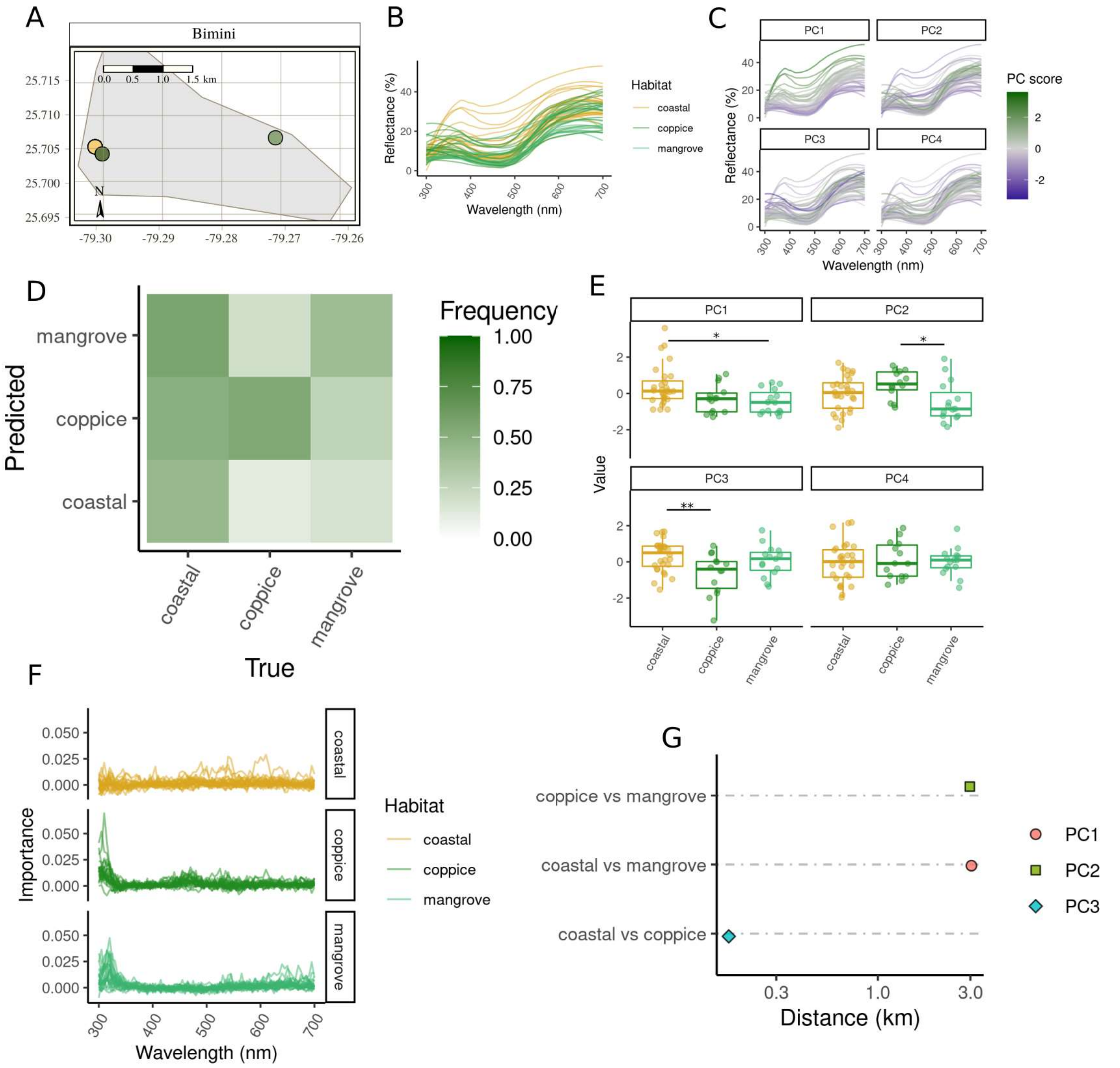

Figure S3: Comparison of dewlap coloration across habitats on Bimini. Legend is as per Figure S2.

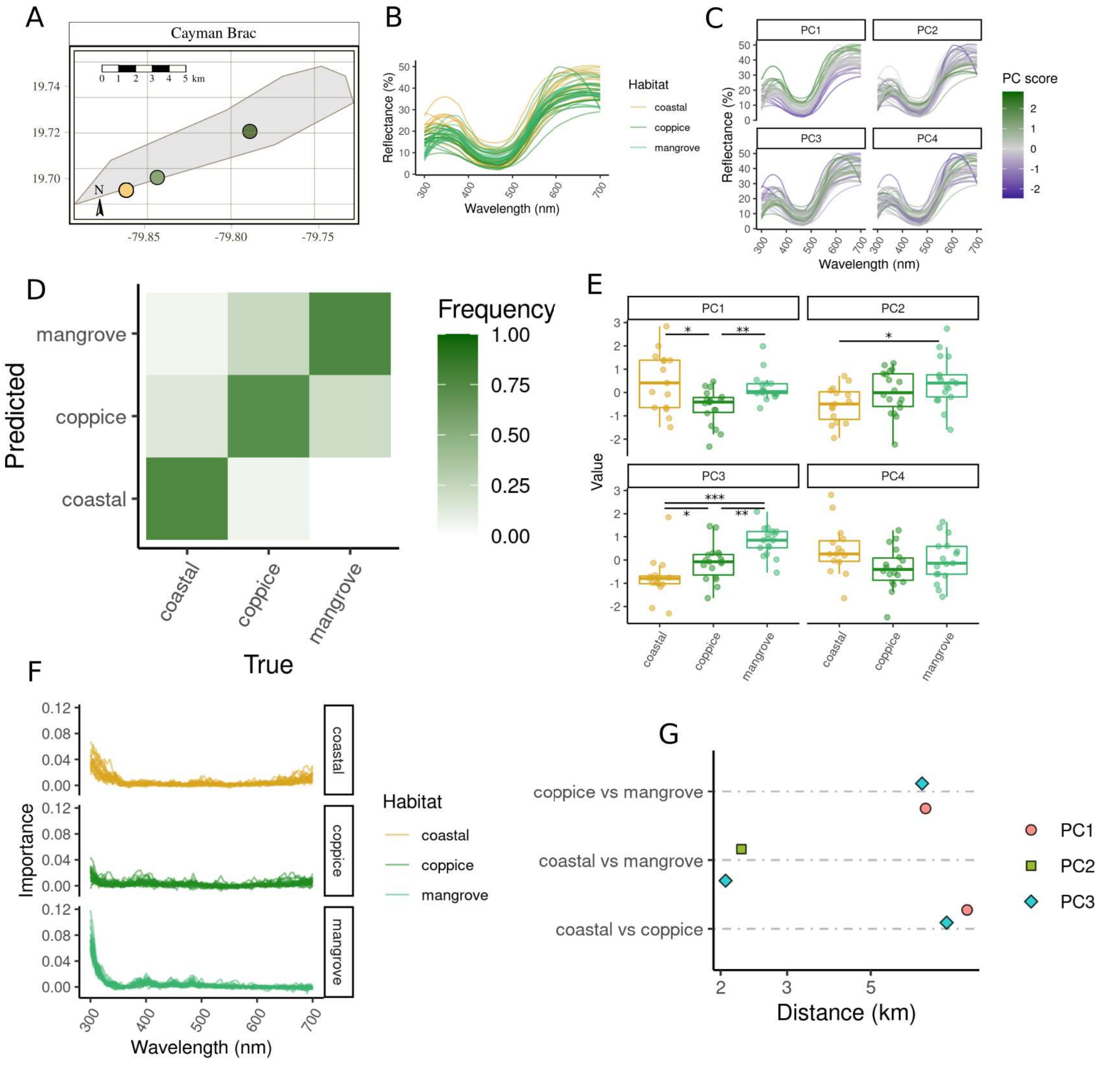

Figure S4: Comparison of dewlap coloration across habitats on Cayman Brac. Legend is as per Figure S2.

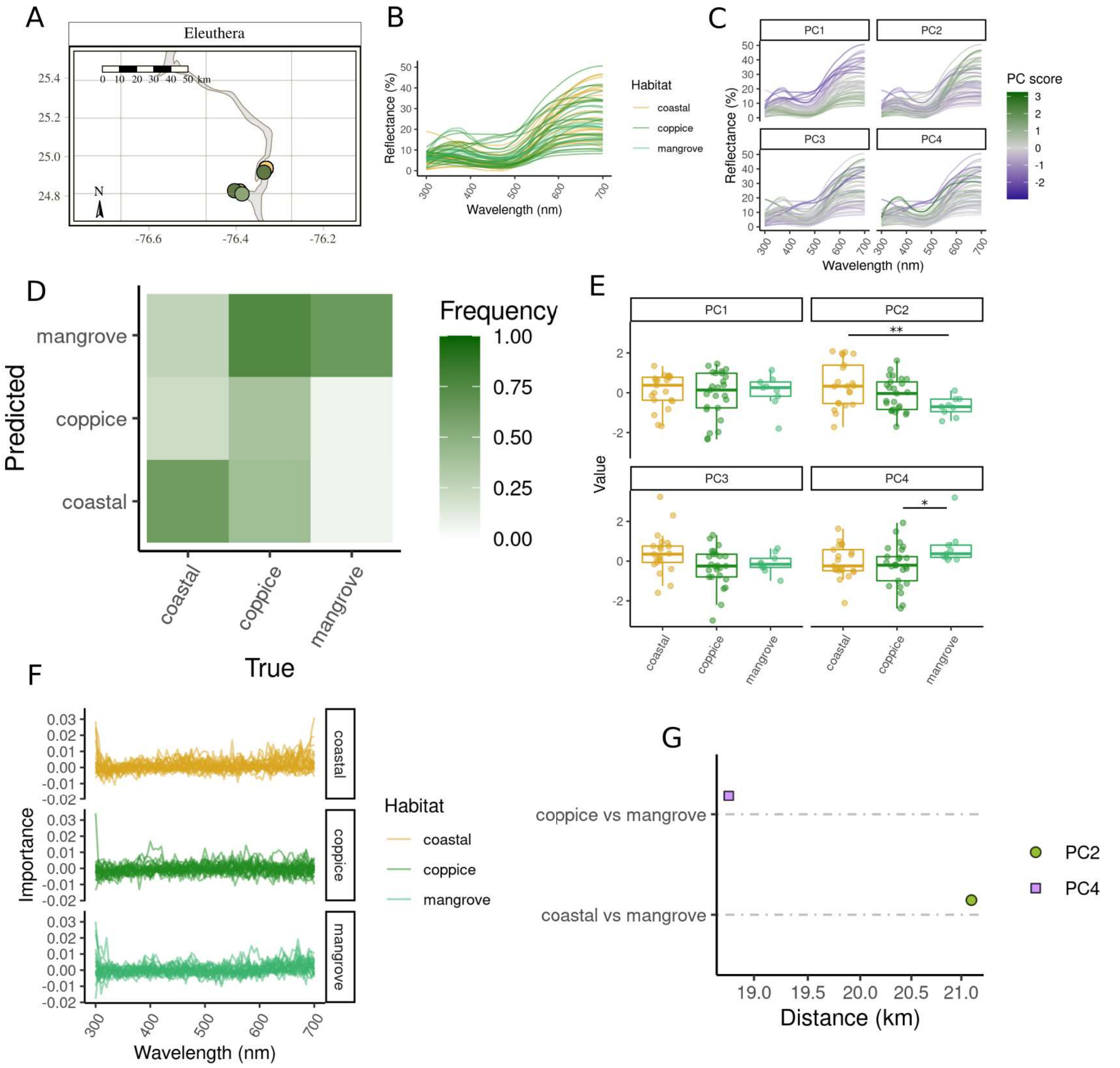

Figure S5: Comparison of dewlap coloration across habitats on Eleuthera. Legend is as per Figure S2.

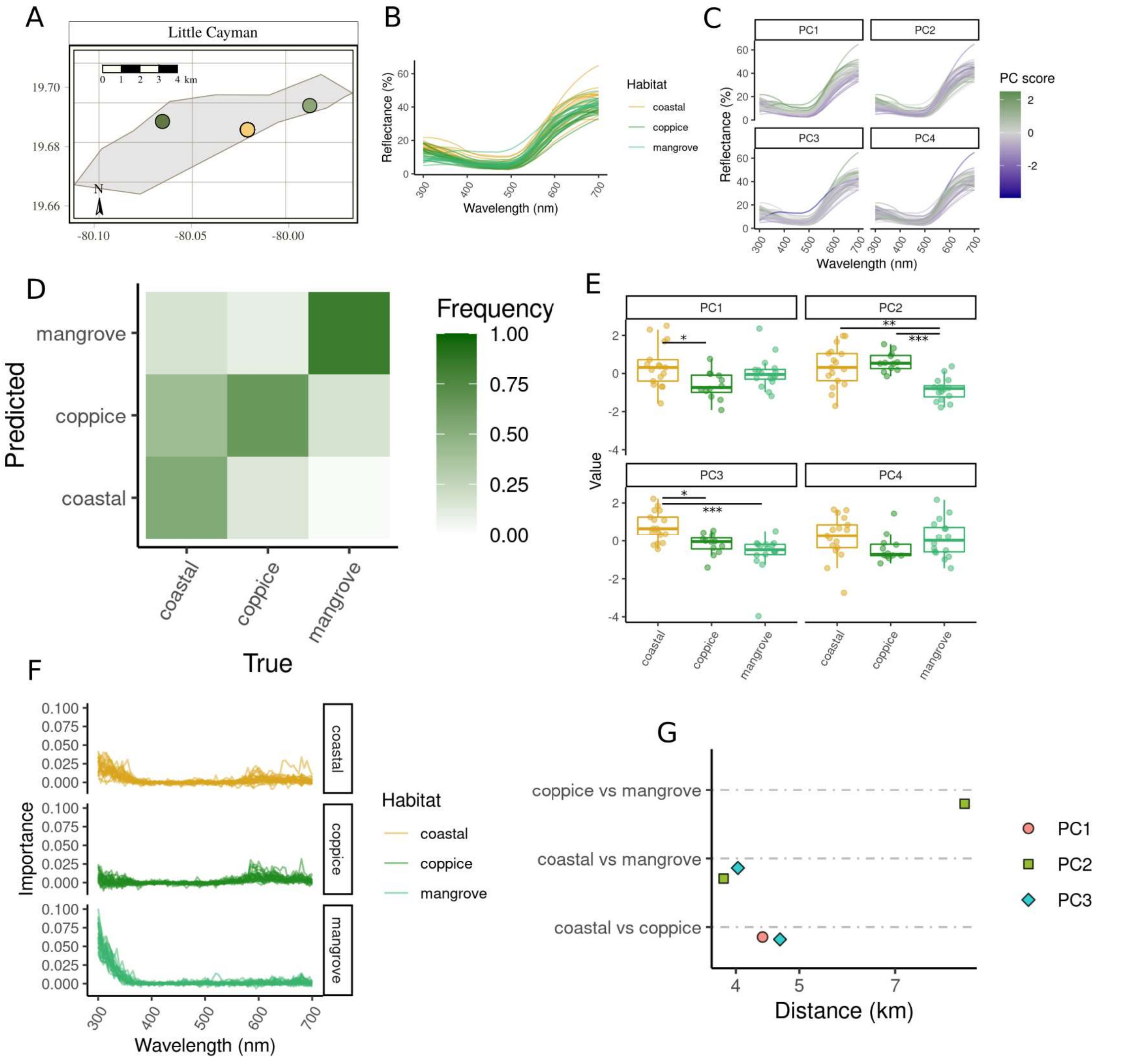

Figure S6: Comparison of dewlap coloration across habitats on Little Cayman. Legend is as per Figure S2.

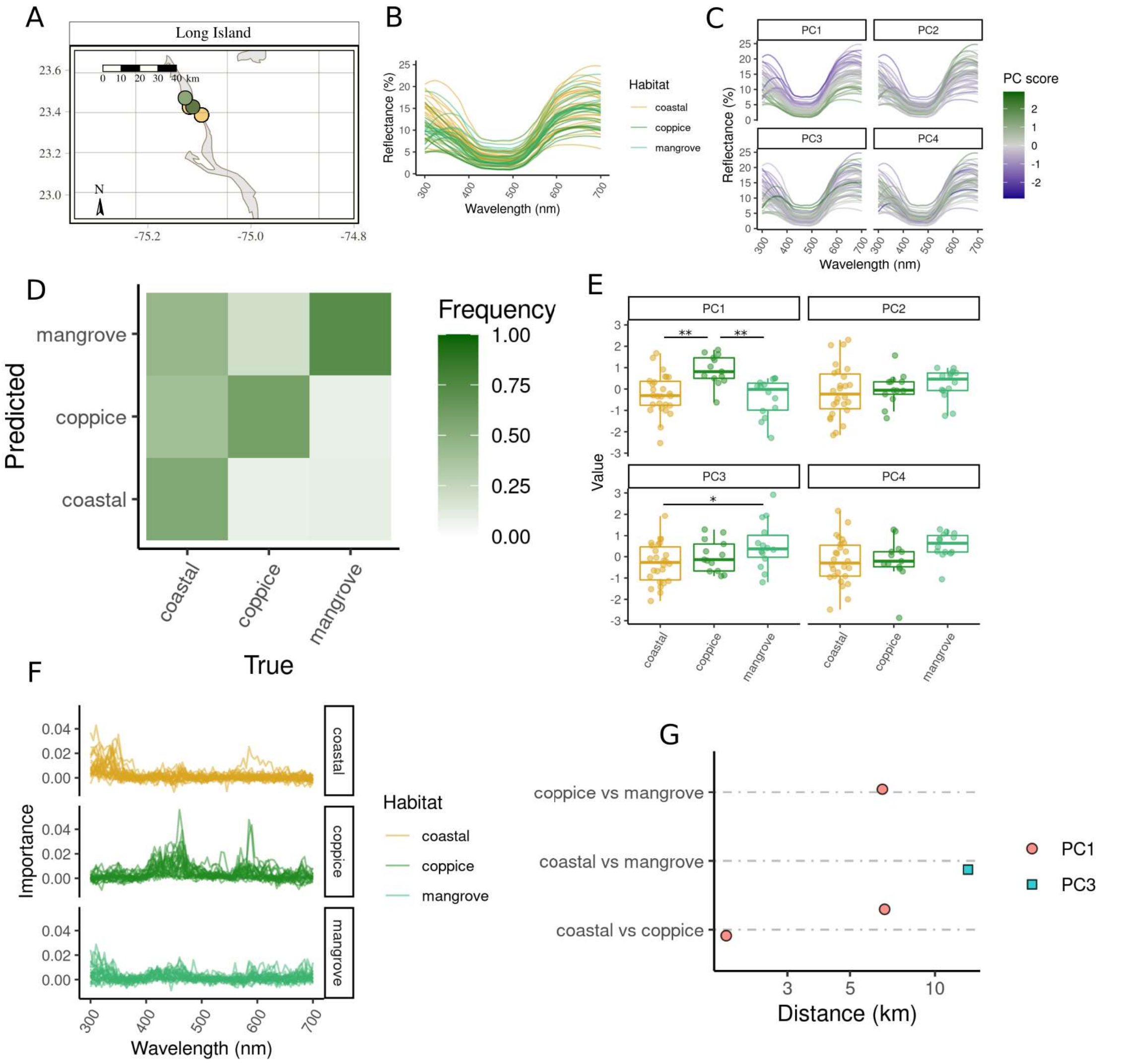

Figure S7: Comparison of dewlap coloration across habitats on Long Island. Legend is as per Figure S2.

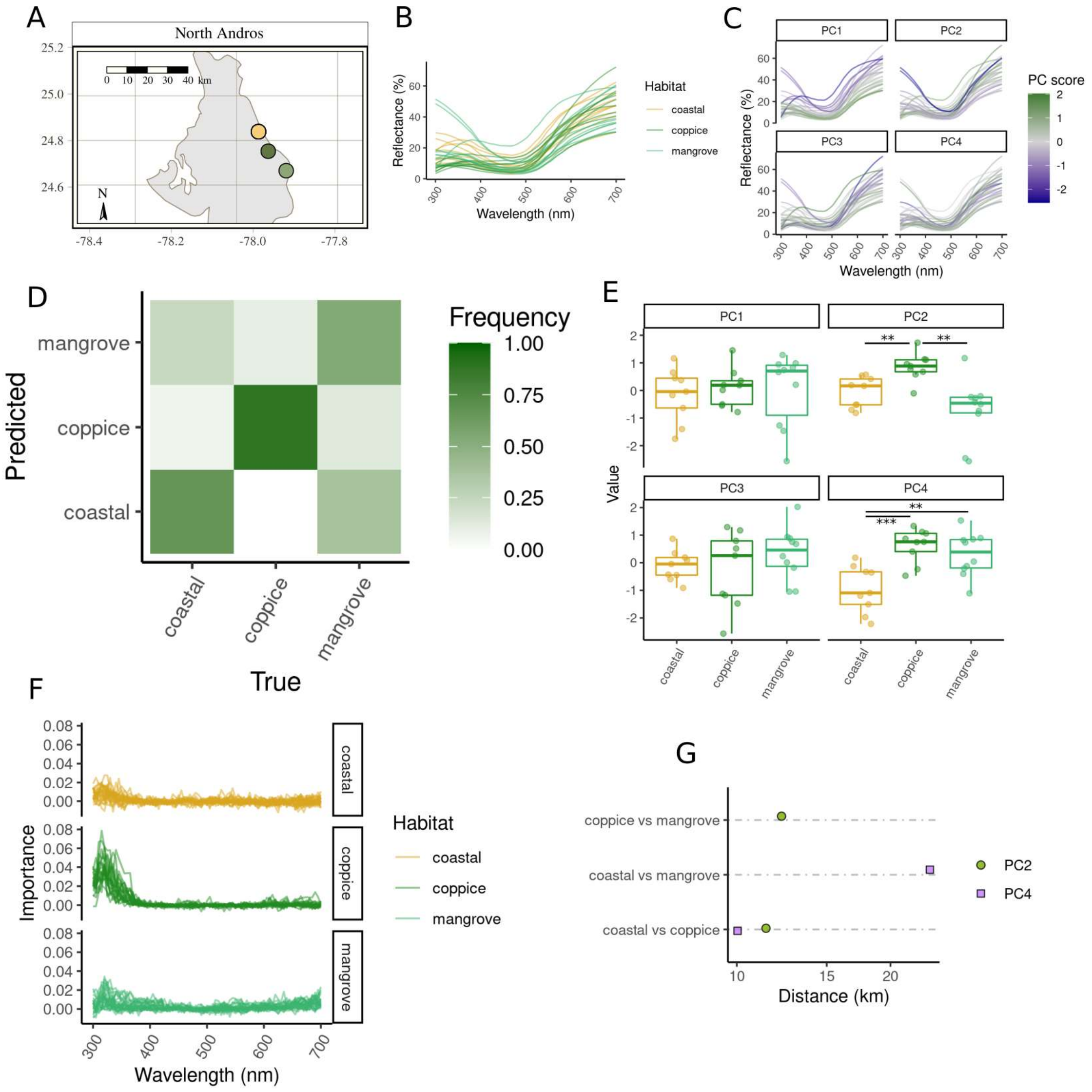

Figure S8: Comparison of dewlap coloration across habitats on North Andros. Legend is as per Figure S2.

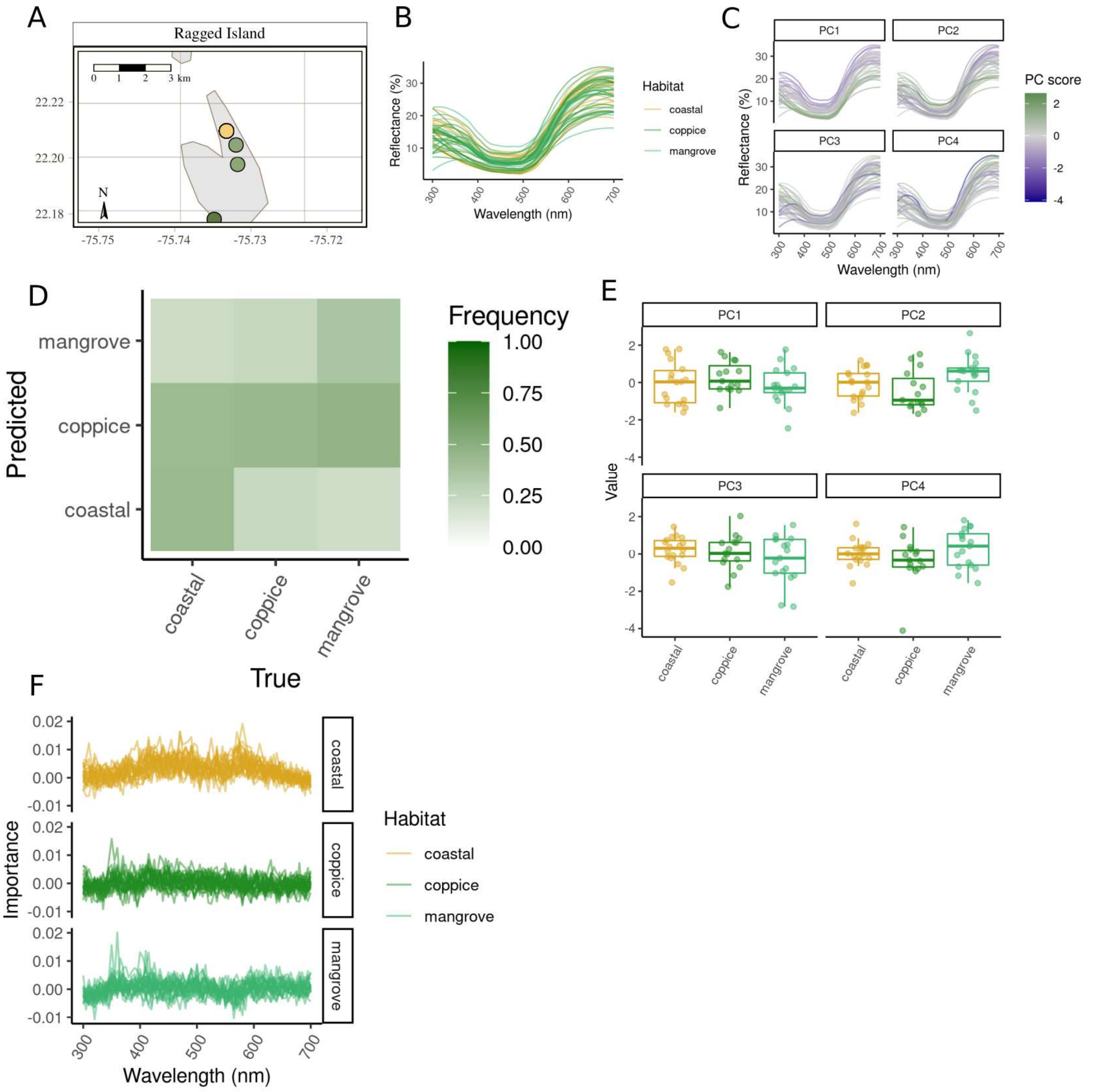

Figure S9: Comparison of dewlap coloration across habitats on Ragged Island. Legend is as per Figure S2, but without panel G.

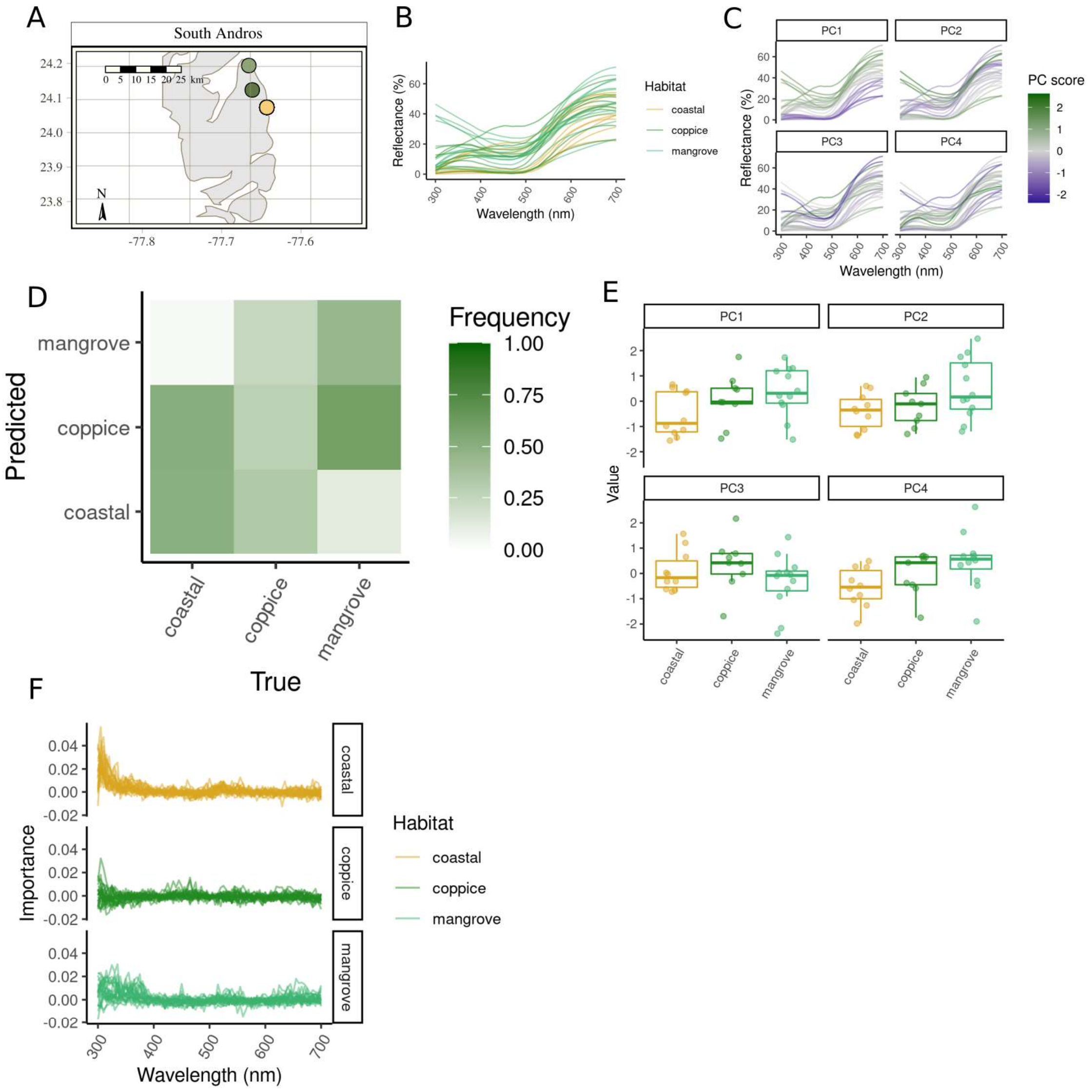

Figure S10: Comparison of dewlap coloration across habitats on South Andros. Legend is as per Figure S2, but without panel G.

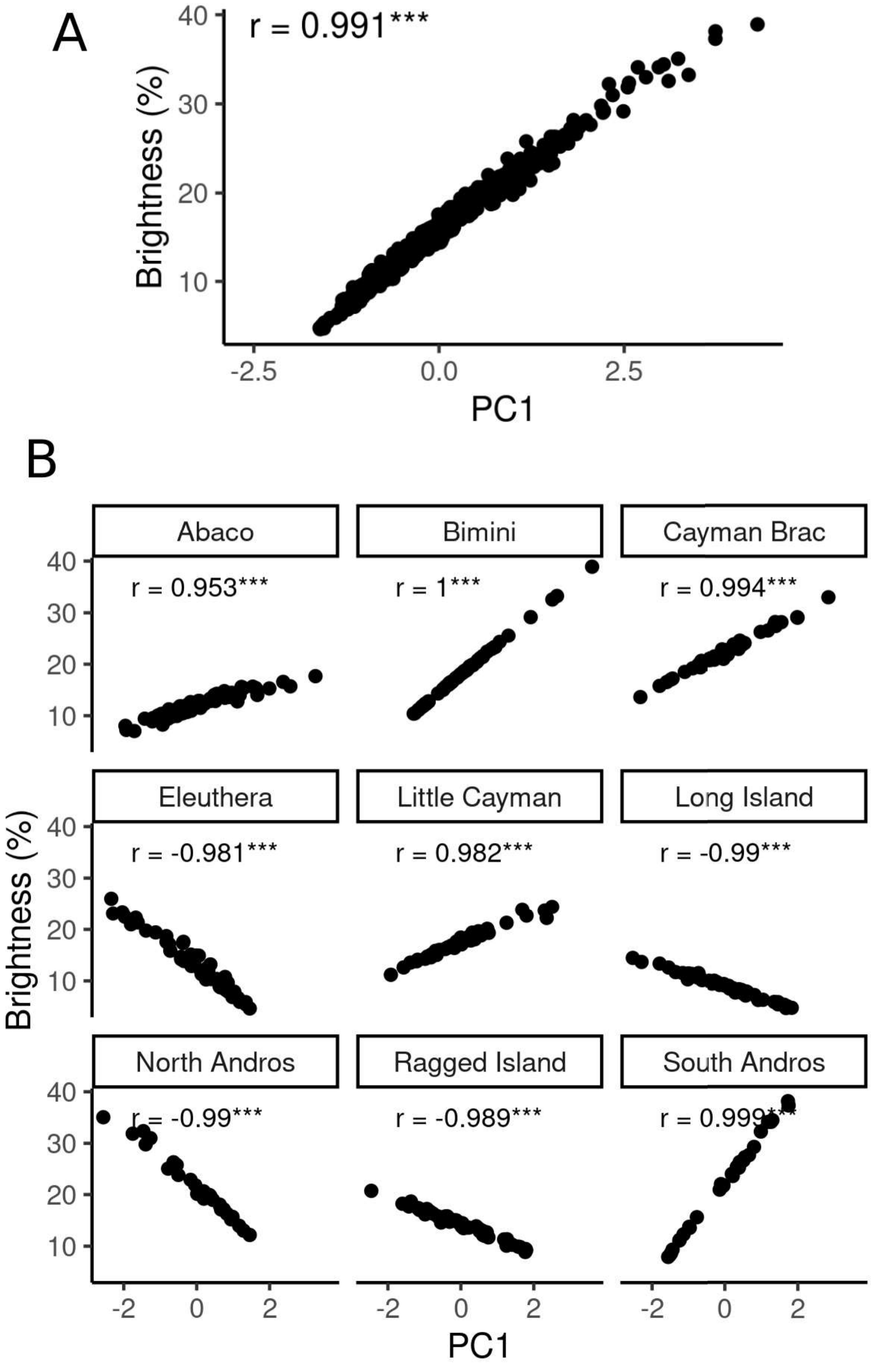

Figure S11: PC1 captures brightness across all islands. (A) Correlation between dewlap brightness (as measured by the mean reflectance from 300 to 700nm in wavelength) and PC1 score across all islands. (B) Correlation between brightness and within-island PC1, for each island. Pearson's correlation coefficients are reported. ***, $P < 0.001$.

Supplementary Tables

Table S1: Number of lizards sampled in each habitat on each island.

	coastal	coppice	mangrove
Abaco	41	24	21
Bimini	28	14	15
Cayman Brac	15	18	17
Eleuthera	21	25	9
Little Cayman	17	12	16
Long Island	26	13	14
North Andros	9	9	10
Ragged Island	18	15	17
South Andros	10	9	12

Table S2: Proportion of variance explained by the first four principal components on each island, as well as in the PCA performed on all islands together (last row).

Island	Total	PC1	PC2	PC3	PC4
Abaco	0.906	0.400	0.277	0.149	0.079
Bimini	0.919	0.530	0.171	0.166	0.052
Cayman Brac	0.888	0.438	0.190	0.155	0.105
Eleuthera	0.926	0.486	0.235	0.138	0.066
Little Cayman	0.907	0.441	0.212	0.176	0.078
Long Island	0.916	0.501	0.215	0.152	0.048
North Andros	0.952	0.485	0.237	0.169	0.062
Ragged Island	0.907	0.483	0.226	0.127	0.072
South Andros	0.937	0.559	0.172	0.151	0.056
All islands	0.915	0.482	0.189	0.167	0.077

Table S3: Henze-Zirkler's test of multivariate normality, performed on global principal components (i.e. fitted on data from all islands together) in each habitat and on each island. The number of outlier points detected based on the Mahalanobis distance is reported. HZ, test statistic. *, $P < 0.05$; **, $P < 0.01$; ***, $P < 0.001$.

Island	Habitat	Outliers	HZ	P	
Abaco	coastal	0	1.097	0.0028	**
Abaco	coppice	0	1.091	0.0016	**
Abaco	mangrove	0	1.052	0.0028	**
Bimini	coastal	0	0.965	0.0199	*
Bimini	coppice	0	0.828	0.0667	
Bimini	mangrove	0	1.211	< 0.0001	***
Cayman Brac	coastal	0	0.648	0.5261	
Cayman Brac	coppice	0	0.691	0.4287	
Cayman Brac	mangrove	0	0.671	0.4838	
Eleuthera	coastal	0	1.446	< 0.0001	***
Eleuthera	coppice	0	1.479	< 0.0001	***
Eleuthera	mangrove	0	0.592	0.5556	
Little Cayman	coastal	0	0.626	0.6547	
Little Cayman	coppice	0	0.637	0.4895	
Little Cayman	mangrove	0	0.867	0.0454	*
Long Island	coastal	0	0.834	0.1291	
Long Island	coppice	0	1.001	0.0034	**
Long Island	mangrove	0	0.907	0.0197	*
North Andros	coastal	0	0.747	0.1123	
North Andros	coppice	0	0.644	0.3623	
North Andros	mangrove	0	0.839	0.0345	*
Ragged Island	coastal	0	0.757	0.2233	
Ragged Island	coppice	0	0.810	0.0944	
Ragged Island	mangrove	0	0.524	0.9341	
South Andros	coastal	0	0.722	0.1765	
South Andros	coppice	0	0.628	0.4212	
South Andros	mangrove	0	0.684	0.3250	

Table S4: Shapiro-Wilk's test of univariate normality of the standardized residuals from OLS and GLS-ANOVAs performed on each island where significant differences were detected by random forest classification. W, test statistic. *, $P_{adj} < 0.05$; **, $P_{adj} < 0.01$; ***, $P_{adj} < 0.001$.

Island	Variable	W	P	P_{adj}	
Abaco	PC1	0.962	0.0128	0.0599	
Abaco	PC2	0.960	0.0093	0.0518	
Abaco	PC3	0.982	0.2963	0.6455	
Abaco	PC4	0.983	0.3168	0.6455	
Bimini	PC1	0.918	0.0009	0.0125	*
Bimini	PC2	0.985	0.7104	0.7956	
Bimini	PC3	0.976	0.2997	0.6455	
Bimini	PC4	0.977	0.3529	0.6455	
Cayman Brac	PC1	0.986	0.8236	0.8869	
Cayman Brac	PC2	0.989	0.9299	0.9299	
Cayman Brac	PC3	0.934	0.0079	0.0518	
Cayman Brac	PC4	0.981	0.5926	0.7214	
Eleuthera	PC1	0.936	0.0058	0.0518	
Eleuthera	PC2	0.972	0.2199	0.6455	
Eleuthera	PC3	0.981	0.5224	0.6965	
Eleuthera	PC4	0.977	0.3689	0.6455	
Little Cayman	PC1	0.955	0.0821	0.3094	
Little Cayman	PC2	0.982	0.6854	0.7956	
Little Cayman	PC3	0.891	0.0005	0.0125	*
Little Cayman	PC4	0.977	0.4858	0.6965	
Long Island	PC1	0.978	0.4223	0.6956	
Long Island	PC2	0.980	0.5161	0.6965	
Long Island	PC3	0.981	0.5605	0.7134	
Long Island	PC4	0.976	0.3580	0.6455	
North Andros	PC1	0.936	0.0884	0.3094	
North Andros	PC2	0.966	0.4735	0.6965	
North Andros	PC3	0.982	0.8977	0.9299	
North Andros	PC4	0.959	0.3348	0.6455	

Table S5: Locations of the sampling sites across islands, with mean within-island principal component scores per site.

Island	Longitude	Latitude	Habitat	PC1	PC2	PC3	PC4
Abaco	-77.7	26.9	mangrove	1.005	-0.116	0.063	-1.255
Abaco	-77.6	26.9	coastal	0.157	-0.531	-0.086	-2.034
Abaco	-77.6	26.9	coppice	-0.081	-0.034	-0.631	-0.633
Abaco	-77.2	26.1	coastal	0.296	0.091	0.987	-0.158
Abaco	-77.0	26.3	mangrove	-0.312	1.224	-0.898	0.208
Abaco	-77.0	26.3	coppice	0.094	-0.314	-0.120	0.482
Abaco	-77.0	26.3	coastal	-0.334	-0.748	0.201	0.534
Bimini	-79.3	25.7	coastal	0.399	0.000	0.304	-0.049
Bimini	-79.3	25.7	coppice	-0.299	0.511	-0.657	0.097
Bimini	-79.3	25.7	mangrove	-0.466	-0.477	0.046	0.000
Cayman Brac	-79.9	19.7	coastal	0.483	-0.523	-0.781	0.443
Cayman Brac	-79.8	19.7	mangrove	0.219	0.453	0.810	-0.015
Cayman Brac	-79.8	19.7	coppice	-0.610	0.008	-0.114	-0.355
Eleuthera	-76.3	24.8	coppice	0.170	-0.855	-0.042	0.086
Eleuthera	-76.3	24.8	coastal	0.503	-0.653	0.564	-0.587
Eleuthera	-76.3	24.8	mangrove	0.067	-0.694	-0.119	0.718
Eleuthera	-76.2	24.9	coppice	-0.353	0.595	-0.544	-0.535
Eleuthera	-76.1	24.9	coastal	-0.215	1.220	0.297	0.396
Little Cayman	-80.1	19.7	coppice	-0.621	0.614	-0.179	-0.404
Little Cayman	-80.0	19.7	coastal	0.395	0.355	0.743	0.175
Little Cayman	-80.0	19.7	mangrove	0.047	-0.838	-0.655	0.118
Long Island	-75.2	23.5	mangrove	-0.390	0.217	0.541	0.535
Long Island	-75.2	23.4	coastal	0.038	-0.807	0.015	-0.326
Long Island	-75.2	23.4	coppice	0.894	0.018	0.035	-0.161
Long Island	-75.1	23.4	coastal	-0.408	0.299	-0.512	-0.134
North Andros	-77.9	24.8	coastal	-0.152	-0.074	-0.104	-0.957
North Andros	-77.8	24.8	coppice	0.112	0.860	-0.258	0.621
North Andros	-77.8	24.7	mangrove	0.036	-0.707	0.326	0.303
Ragged Island	-75.7	22.2	coppice	0.269	-0.440	0.088	-0.360
Ragged Island	-75.7	22.2	coastal	-0.049	-0.076	0.221	0.029
Ragged Island	-75.7	22.2	mangrove	-0.246	0.273	-0.118	0.933
Ragged Island	-75.7	22.2	mangrove	-0.099	0.749	-0.588	-0.636
South Andros	-77.6	24.2	mangrove	0.384	0.492	-0.333	0.472
South Andros	-77.6	24.1	coppice	0.067	-0.211	0.358	-0.018
South Andros	-77.5	24.1	coastal	-0.520	-0.401	0.078	-0.550

Table S6: Nonparametric Kruskal-Wallis tests performed on each variable on each island where deviations from normality were detected. *, $P < 0.05$; **, $P < 0.01$; ***, $P < 0.001$.

Island	Variable	χ^2	df	P	
Bimini	PC1	9.31	2	0.0095	**
Little Cayman	PC3	19.95	2	< 0.0001	***

Table S7: Support vector machine classification results. Legend is as per Table 1.

Island	N	Score	P	
Abaco	86	0.614	< 0.0001	***
Bimini	57	0.474	0.0098	**
Cayman Brac	50	0.732	< 0.0001	***
Eleuthera	55	0.476	0.0112	*
Little Cayman	45	0.724	< 0.0001	***
Long Island	53	0.623	< 0.0001	***
North Andros	28	0.721	< 0.0001	***
Ragged Island	50	0.368	0.2874	
South Andros	31	0.445	0.1152	

Table S8: Linear discriminant analysis classification results. Legend is as per Table 1.

Island	N	Score	P	
Abaco	86	0.670	< 0.0001	***
Bimini	57	0.491	0.0046	**
Cayman Brac	50	0.736	< 0.0001	***
Eleuthera	55	0.480	0.0112	*
Little Cayman	45	0.747	< 0.0001	***
Long Island	53	0.562	0.0004	***
North Andros	28	0.636	0.0008	***
Ragged Island	50	0.388	0.1964	
South Andros	31	0.458	0.0589	
